# In-Depth Observation on the Microbial and Fungal Community Structure of Four Contrasting Tomato Cultivation Systems in Soil Based and Soilless Culture Systems

**DOI:** 10.3389/fpls.2020.520834

**Published:** 2020-11-05

**Authors:** Oliver Grunert, Emma Hernandez-Sanabria, Saskia Buysens, Stefaan De Neve, Marie-Christine Van Labeke, Dirk Reheul, Nico Boon

**Affiliations:** ^1^Greenyard, Skaldenstraat 7a, Desteldonk, Belgium; ^2^Agaris, Desteldonk, Belgium; ^3^VIB – KU Leuven Center for Microbiology, Laboratory of Molecular Bacteriology, Rega Institute Herestraat, Leuven, Belgium; ^4^PCG – Vegetable Research Centre, Kruishoutem, Belgium; ^5^Department of Environment, Faculty of Bioscience Engineering, Ghent University, Ghent, Belgium; ^6^Department of Plants and Crops, Faculty of Bioscience Engineering, Ghent University, Ghent, Belgium

**Keywords:** tomato, soilless culture systems, growing medium, soil, microbial community, malt sprouts, aquaponics, organic fertilizer

## Abstract

As soil and soilless culture systems are highly dynamic environments, the structure of rhizosphere microbial communities is consistently adapting. There is a knowledge gap between the microbial community structure of soil based and soilless culture systems and thus we aimed at surveying their impact on diversity and composition of bacterial communities across a 10-month period in a tomato cultivation system. We compared community metrics between an soil based culture system fertilized with malt sprouts and blood meal, known for its slow and high mineralization rate, respectively and a soilless culture system fertilized with fish effluent or supplemented with an liquid organic fertilizer. Bacterial and fungal community composition was followed over time using two complementary techniques, phospholipid fatty acid analysis and 16S rRNA amplicon sequencing. Nitrogen dynamics and plant performance were assessed to provide insight on how bacterial diversity of soil and soilless microbial communities ultimately impacts productivity. Similar plant performance was observed in soilless culture systems and soil based system and yield was the highest with the aquaponics-derived fertilizer. Soil and soilless cultivating systems supplemented with different nitrogen-rich fertilizers differed on its characteristics throughout the experimental period. Fast-paced fluctuations in pH(H_2_O) and nutrient cycling processes were observed in growing medium. Physicochemical characteristics changed over time and interacted with bacterial community metrics. Multivariate analysis showed that plant length, pH, *Flavisolibacter*, phosphorus, chloride, ammonium, potassium, calcium, magnesium, sodium, electrical conductivity, nitrate, sulfate, and the bacterial genera *Desulfotomaculum*, *Solirubrobacter*, *Dehalococcoides*, *Bythopirellula*, *Steroidobacter*, *Litorilinea*, *Nonomuraea* were the most significant factors discriminating between natural soils supplemented with animal and plant by-products. Long-term fertilizer regimes significantly changed the PLFA fingerprints in both the soilless culture and soil based culture system. The use of these by-products in the soil was positively associated with arbuscular mycorrhizal fungi (AMF), which may influence rhizosphere communities through root exudates and C translocation. Community structure was distinct and consistently different over time, despite the fertilizer supplementation. The fungal microbial community composition was less affected by pH, while the composition of the bacterial communities (Actinomycetes, Gram-negative bacteria, and Gram-positive bacteria) was closely defined by soil pH, demonstrating the significance of pH as driver of bacterial community composition. Fertilizer application may be responsible for variations over time in the ecosystem. Knowledge about the microbial interactions in tomato cultivating systems opens a window of opportunity for designing targeted fertilizers supporting sustainable crop production.

## Introduction

Over the past century, incredible advancement has been achieved worldwide in increasing global agricultural production (Ramankutty et al., 2018) The production has more than tripled between 1960 and 2015, owing predominantly to the Green Revolution technologies and a significant enlargement in the use of land, water and other natural resources for agricultural purposes (FAO, 2016; Ramankutty et al., 2018). In order to meet the agricultural demand in 2050 we will need to produce 50% more food (Alexandratos, 2009; Foley, 2011; McKenzie and Williams, 2015). This expansive food production comes at a hefty cost to the natural environment (Notarnicola et al., 2017). The urgent need for more sustainability prompted a renewed attention in the biology-based elements of soil and soilless culture crop production systems, including interest in the development of agricultural and horticultural biological solutions. The time has come for another look at using the tools of nature to enhance the intrinsically plant biological systems. This doesn’t implicit an anti-chemical approach: rather, make agricultural practices both more productive and more sustainable by incorporating the next generation of biologically sourced tools into existing practices.

The use of fertilizers facilitated largely the increases in agricultural production over the last decennia. Before the 1950s, farmers used natural fertilizers such as manure (Gellings and Parmenter, 2016) and there was very limited use of chemical fertilizers. Nitrogen (N), phosphorus (P), and potassium (K) are essential for plant growth, and potassium and phosphorus are found in mineral deposits (Roberts and Stewart, 2002; Gross, 2012). A nitrogen revolution was generated by the introduction of the Haber-Bosch process: the industrial scale production of ammonia from natural gas (Smil, 1999). Today, the total fertilizer nutrient demand is more than 190 million tons (∼120 tons N, ∼46 tons P, ∼37 tons K) with an average annual growth of 1.9 percent expected over the following years next to other measures (Heffer and Prud’homme, 2016; FAO, 2017). These inorganic nutrients are instantly usable and the leftover either rises up in the soil, disappeared as run-off into the surface water or drains into the groundwater (Steiner et al., 2007). Avoiding reduction of soil organic carbon (SOC) and too expeditious availability, one can go for organic fertilizers (Diacono and Montemurro, 2011) typically produced from plant, or animal-derived materials (Dion et al., 2020) and even microbes can be used (Verstraete et al., 2016; Pikaar et al., 2017; Sakarika et al., 2019; Spanoghe et al., 2020). Blood meal has been reported to provide a fast and high-percentage N mineralization (Agehara and Warncke, 2005; Hammermeister et al., 2006; Hartz and Johnstone, 2006; Gaskell and Smith, 2007; Sullivan et al., 2010). Plant based materials are reported to be slow-release N fertilizer (Agehara and Warncke, 2005; Hammermeister et al., 2006; Sullivan et al., 2010), which is consistent with its higher C/N ratio. These organic fertilizers are mixed in the growing medium or soil and the breakdown and subsequent rate of nutrient delivery in soil and soilless culture systems largely depends on the physical, chemical and biological characteristics of the soil or soilless culture system (Schmilewski, 2008; Sonneveld and Voogt, 2009; Grunert et al., 2016a).

Besides fertilizers, the fast development in soilless production systems caused a considerable switch away from the use of soil to soilless culture systems and consequently pushed the increase in food production (Raviv et al., 2019). Soilless plant culture is any mechanism of growing plants without the benefit of soil as rooting medium (Schmilewski, 2007; Savvas et al., 2013; Raviv et al., 2019) and they play a pivotal role in horticulture and agriculture (Barrett et al., 2016). The enduring transfer from soil to soilless culture systems is also advanced by a good management of various essential factors, partly responsible for enhanced plant performance (Barrett et al., 2016). Soilless horticultural systems have benefits over soil based systems in that the nutrients (Dubik et al., 1990), oxygen and water required for a healthy plant growth are controlled (Barrett et al., 2016) and that soil-borne pathogens can be avoided (Runia, 1993; Postma, 2009). Soil-based organic culture systems are typified by a combination of low external input methods. It gives next to other measures preference to improving the soil with compost additions and animal and plant derived green manures (Reganold and Wachter, 2016). In addition, organic soil management is inherently dependent on an active bacterial, saprotrophic and fungal community (Reganold and Wachter, 2016; Martínez-García et al., 2018) and has an increased microbial diversity (Francioli et al., 2016). However, resource efficiency and production yields in these soil based organic culture systems are relatively low (Rahmann et al., 2009).

Soil-based and soilless culture systems rely upon physicochemical features, which are distinct for each culture system. Soil and soilless culture systems have diverse physical and hydraulic characteristics (Raviv and Lieth, 2007). Soil-grown plants are encountered with relatively high water availability shortly after fertigation (Bunt, 1988). Another basic trait of soilless cultivation over soil-based cultivation is the boundless root volume, while in soilless culture the root volume is containerized (Raviv et al., 2019). Nutrients, pH and the electrical conductivity (EC) are influential chemical properties (Lauber et al., 2008, 2009, 2013; Raviv et al., 2019) and can be easily regulated in soilless culture systems to demanded nutrient, pH and EC levels. It was demonstrated, that soilless organic growing media have particular niches for diverse bacterial communities with temporal functional stability (Grunert et al., 2016a). New or sterilized growing media usually experience the absence of a diverse and competitive microbiome (Raviv and Lieth, 2007; Postma, 2009; Grunert et al., 2016a), while the soil generally holds up to 10^7^ –10^9^ colony-forming units (CFU) of bacteria and 10^4^ –10^6^ culturable fungal propagules per gram of soil (Alexander, 1977). It is postulated that organic peat based growing media used in tomato cultivation systems are mainly colonized by fungi, actinomycetes and *Trichoderma* spp. (Khalil and Alsanius, 2001; Koohakan et al., 2004), while mineral growing media are mainly colonized by bacteria (Vallance et al., 2010). In addition, changes in the soil water content as a result of fertigation activities greatly impacts microbial activity and community structure (Fierer et al., 2003). Engineering the microbial community of soilless culture systems might help to work out approaches to progress toward a more sustainable horticulture with increased productivity, quality and sustainability. However, little comparative research has been performed on the bacterial and fungal composition and development during crop growth in soil and soilless culture systems supplemented with organic and chemical fertilizers. Thus, improved understanding of the variability over time in soil and soilless microbial communities amended with organic and chemical fertilizers will provide insight into the factors influencing the overall diversity and might help developing advanced soil and soilless culture systems.

The present study used a multidisciplinary approach to study tomato cultivation systems and the overall objective of the research was to carry out an in-depth observation of four contrasting tomato cultivation systems during one growing season on the composition of the bacterial and the fungal microbiome. The objectives of the experiment were twofold. First, the effect of four different tomato cultivation systems on plant performance was studied and second changes in microbial community composition during 321 days after sowing were assessed. Assuming that each of the four contrasting tomato cultivation systems and different nutrient management have an influence on physico-chemical characteristics, we hypothesized that (i) the soil and soilless culture based edaphic properties, which are strongly altered during one tomato growing season, consecutively affect the bacterial and fungal microbial community structure. Moreover, we hypothesized that (ii) the community changes caused by four contrasting fertilization strategies included shifts in the abundance of various plant-beneficial soil- and soilless culture based microorganisms, thus influencing plant performance.

## Materials and Methods

### Experimental Setup for Soil-Based and Soilless Culture System

#### Four Contrasting Tomato Cultivation Systems

Four contrasting tomato cultivation systems were used, i.e., two soilless grow bag (GB) based and two soil-based culture systems (SOIL). The GB were filled with an organic growing medium made of 40% v/v sod peat, 40% v/v Irish peat and 20% v/v coconut fiber. The compartment for the soilless culture system was split in two sections. In the first section liquid organic fertilizer solutions (GBOF) through the fertigation systems, while in the second section plants were fertilized with fish effluent supplemented with mineral fertilizer (GBFISH). The soil (SOIL) used for the two soil-based tomato cultivating systems at the experimental site (PCG Kruishoutem, Belgium) is an organically managed soil according to the EU Council Regulation 834/2007. For most of the European countries and for all member states of the European Union (EU), organic farming is strictly defined by the European Commission (EC) and these rules were followed for the soil based system. The soil had a loamy sand texture (Haplic Podzol: 85% sand, 11% silt, and 4% clay). For the organic soil two different kinds of fertilizers were used: plant-derived malt sprouts (3-0-0) (Orgamé, Belgium) material (SOIL-PLANT) and animal-derived blood meal (14-0-0) (Orgamé, Belgium) material (SOIL-ANIMAL). The malt sprouts and the blood meal were blended in the soil on 19/3/2015, 2/7/2015 and 22/7/2015 and 28/8/2015.

#### Experimental Setup of the Four Tomato Cultivation Systems

**FIGURE 1:**
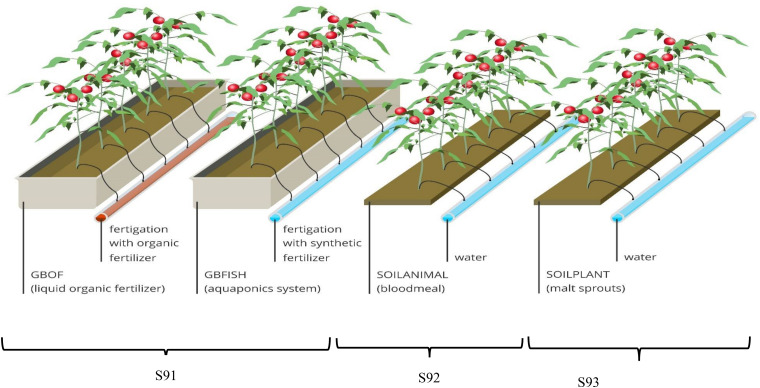
Overview of the experimental set-up showing one experimental unit per treatment. The glass house was divided into three compartments (S91, S92, and S93) with a surface of 80 m^2^ for each compartment. The compartment for the soilless culture system (S91) was subdivided into two part. i.e., GBOF and GBFISH; S92 was organic soil fertilized with animal (blood meal) derived material and S92 was the organic soil fertilized with plant-derived material (malt sprouts). In S92 and S93 the previous cultures were tomato in 2014, pepper in 2013 and cucumber in 2012. Plant density was the same for all the treatment 2.65 plants m^– 2^. For the soilless culture system and soil-based system five experimental units were randomly selected from the different compartments.

The growth tests for the two soilless culture systems (GBOF and GBFISH) were performed in the same compartment (S91), while the soil-based tomato cultivation system were performed in two different compartments (S92 and S93). For the soilless culture system, GBs or slabs were placed in gutters (or gullies) to collect the efflux solution typically called ‘drain,’ which was discharged. Forty one-headed tomato plants per treatment were used (GBOF, GBFISH, SOILPLANT, and SOILANIMAL) and each tomato cultivation systems had an effective experimental surface of 15.1 m^2^ per treatment out of the available 80 m^2^ per compartment. Slabs for the soilless culture system were placed in gutters and each gutter contained 6 slabs. Slabs of GB had the following dimensions: 1.0 m × 0.2 m × 0.085 m. The mature plants (5 plants per slab) with a visible first truss were placed on the slabs in the soilless culture system and directly on the soil in the soil based system (Figure 1). This was done on the same date (55 DAS). Plant density was 2.65 plants m^–2^ (i.e. 1 plant per 0.47 m × 0.8 m) and was equal among treatments. The high-wire system was used for the tomato cultivation. In this system the growing tip remained at the top of the canopy and the stem was lowered allowing maximum light interception of the head of the plant. The plants grown in the organically managed soil had about 113 L soil plant ^–1^ at their disposal, assuming that the plants used between 0 and 30 cm of the top layer for rooting, while the plants in the soilless culture system had about 3.4 L growing medium plant^–1^ at their disposal. For sampling only the inner plants were used to avoid border effects. The first harvest started on 138 DAS and ended on 321 DAS. Water gift was registerend for all the tomato cultivating systems (Supplementary Figure S2). All guidelines according to organic greenhouse tomato production, with respect to organic fertilizers and pesticides use, were followed. Side shoots developed from every axil and these side shoots were removed on a weekly basis leaving only one main stem as a growing point. Uniform fruit size was maintained by fruit pruning and thereby controlling the number of fruits left on the truss, i.e. 5 fruits per truss.

#### Tomato Plants and Transplant Production

Tomato plants (*Solanum lycopersicum* cultivar RZ 72-704, Rijk Zwaan, Fijnaart, Netherlands) was grafted on *Solanum lycopersicum* L. x *Solanum habrochaites* Maxifort (Monsanto Vegetable Seeds, Bergschenhoek, Netherlands). Tomatoes were sown on 18/12/2014 (0 DAS) and transplant production of grafted plants required up to 6 weeks. Transplant production was done in blocking compost made of white and black peat according to EU Council Regulation 834/2007. The blocking compost had the following dimensions:0.1 m × 0.1 m × 0.06 m. The tomato plants were grown until mature plants (5 plants per slab) with a visible first truss and which was as wide as it was tall.

#### Experimental Location

The plant experiment was established in the experimental glasshouse of the Vegetable Research Centre in Kruishoutem (longitude = 3°31′E and latitude = 50°56′N and 10 meters above sea level). The glasshouse was divided into three compartments (S91, S92, and S93) and every compartment had a surface of 80 m^2^ with equal climatic conditions. Compartment S91 was used for both soilless culture systems and S92 and S93 was used for the soil based tomato cultivation system. An overview of the experimental set-up can be found in Figure 1. The glasshouse experiment started on the 11th of February 2015 and ended on the 4th of November 2015. *Macrolophus pygmaeus* was used to protect the plants against all kind of insects, Enermix (*Encarsia formosa* + *Eretmocerus eremicus*) was used against white flies. *Trichoderma harzanium* (Koppert, Netherlands) is a biolgical fungicide and used to protect the plants against soil borne diseases, such as *Pythium* spp., *Rhizoctonia* spp., *Fusarium* spp., *Sclerotinia* and the root mat syndrome caused by *Agrobacterium rhizogenes.* Serenade (*Bacillus amyloliquefaciens*) from Bayer Crop Science (Germany) and magnesium sulfate were used to protect the plants against *Oidium lycopersici*.

### Irrigation and Nutrient Management of the Four Different Tomato Cultivating Systems

Irrigation of tomatoes was based on solar irradiation and considered amount of drained water. Irrigation varied between 3 mL J^–1^ and 4 mL J^–1^ at higher temperatures resulting in a peak water supply between 8 and 10 L m^–2^ d^–1^ in summer. For the soilless culture system (GBOF and GBFISH) the efflux solution or drain was discharged. It was estimated at the start of the experiment that about 1300 L m^–2^ water was needed for the cultivation of tomatoes during a whole season (February until November). For the four contrasting tomato cultivation systems drip irrigation was used and the nutrients were injected into the irrigation water from concentrated solutions in stock tanks (Sonneveld and Voogt, 2009). For the soil-based tomato cultivation systems (SOILANIMAL and SOILPLANT) no nutrients were infused into the irrigation water as they were blended in the soil, meaning that pure rain water was used (Figure 1). For the soilless culture system slabs may be watered up to 6 times per hour in peak radiation, and up to 30 times per day under summer conditions.

#### Nutrient Management for the Soilless Culture System (GBOF and GBFISH)

The compartment for the soilless culture system was split in two parts. One part was fertilized with a liquid organic fertilizer solution (GBOF) and another part was fertilized with fish effluent supplemented with mineral fertilizer (GBFISH). Four different organic fertilizers (ANTYS MgS, Biosyr, Nutrikali, and SP; Frayssinet, France) were used for GBOF. Detailed information about the type, composition and the amount of fertilizer used can be found in Supplementary Table S4.

They were combined with each other aiming at a balanced nutrient solution suitable for the cultivation of tomatoes. Moreover, a N:P:K ratio of 1:0.2:1.6 was respected throughout the whole experimental period for the soilless culture system. Nutrient solution for GBOF was supplemented with extra calcium chloride (CaCl_2_) and Libremix (3.2% Fe-EDTA, 1.5% Mn-EDTA, 1.6% Cu-EDTA, 0.6% Zn-EDTA, 0.8% B and 2.5% Mo; Brinkman, Netherlands) if needed, such as increased incidence of blossom end rot (BER). The nitrogen dose of the nutrient solution was increased or decreased according to the growth of the plants and/or the presence or absence of deficiency symptoms.

For GBFISH the fish effluent coming from the aquaponics system was supplemented with mineral fertilizer (GBFISH). Ammonia is the main excretion product of the fish. The excreted ammonium was converted into nitrate and was used as the primary inorganic nitrogen source for the tomato plants. The fertigation solution coming from the aquaponic system was amended with the necessary nutrients and corrected when needed aiming at a final composition of 0.7 mmol NH_4_^+^ L^–1^, 18.4 mmol NO_3_^–^ L^–1^, 10.9 mmol K L^–1^, and 6.2 mmol Ca L^–1^, 2.8 mmol Mg L^–1^, 0.7 mmol Cl L^–1^, 5.1 mmol SO_4_^2–^ L^–1^ and 1.7 mmol H_2_PO_4_^–^ L^–1^.

Nitrogen supply rate per square meter increased steadily from 1.3 g N m^–2^ d^–1^ to 112.6 g N m^–2^ d^–1^ between days 78 and 161 for GBOF. Nitrogen load was decreased to 20 g N m^–2^ d^–1^ in the following next 27 days as a result of increased blossom end rot (BER) incidence, development of smaller leaves, reduced plant growth, and leaf chlorosis. Nitrogen supply rate was increased again up to 164 g N m^–2^ d^–1^ after the above mentioned period.

Nutrient management for the soil-based culture system SOILANIMAL treatment received 252 kg N ha^–1^ coming from blood meal with a total nitrogen content of 14% and 1630 kg ha^–1^ of patentkali (30% K_2_O, 10% MgO, and 42% SO_3_). The SOILPLANT treatment received 300 kg N ha^–1^ coming from malt sprouts (7% of nitrogen) and 1630 kg ha^–1^ of patentkali (30% K_2_O, 10% MgO and 42% SO_3_) at the start of the experiment. Chemical composition of the organic soil (SOILPLANT and SOILANIMAL) and the soilless culture system can be found in Table 1. Detailed information about the type, composition and the amount of fertilizer used can be found in Supplementary Tables S2, S3.

**TABLE 1:** Overview of the yield [short culture from May till November, tomatoes with blossom end rot (BER)], the total number tomatoes and the distribution in percentage between loose and tomatoes per vine for four different tomato cultivating systems (GBOF, GBFISH, SOILANIMAL, and SOILPLANT).

Object	Total yield (kg m^–2^)	BER	Tomatoes (number of tomatoes m^–2^)	Loose tomatoes	2 tomatoes per vine	3 tomatoes per vine	4 tomatoes per vine	5 tomatoes per vine	6 tomatoes per vine	7 tomatoes per vine
		%		%	%	%	%	%	%	%
GBOF	22.378	2.8	239	2.1	6.4	17.0	34.0	27.7	8.2	4.6
GBFISH	27.840	0.9	274	0.8	5.2	12.5	22.7	35.8	15.2	7.8
SOILANIMAL	22.501	0	226	3.3	7.2	12.7	24.7	35.0	12.7	4.4
SOILPLANT	24.127	0	224	4.4	5.0	15.6	22.4	37.2	11.9	3.6

### Sample Collection

For the experiment three compartments were used. The compartment for the soilless culture system was split in two parts each consisting of 6 gutters/rows. For an overview of the sampling procedure please check Supplementary Figure S1. For the soil based system two different compartments were used. Slabs for the soilless culture system were placed in gutters, each gutter contained 6 slabs and each slab 5 plants. The two outer rows and outer slabs of each block were not selected, because of possible interactions with the adjacent rows and to avoid side effects. One slab with 5 consecutive plants in the soilless culture system and 5 consecutive plants in the soil based system were considered as an experimental unit. Among all treatments the tomato plants were placed consecutively with an interspacing of 0.47 m and an in row interspacing of 0.8 m. For the soilless culture system and soil-based system five experimental units were randomly selected from the different compartments. Samples of the different experimental units were collected at different time points during the growing season and at the start of the experiment. Ten subsamples from each experimental unit were collected, pooled, homogenized and treated as a single sample. At each time point, samples were taken from 5 fixed experimental units of each GBOF, GBFISH, SOILANIMAL, and SOILPLANT, including root material. Samples from the soil were taken with an auger in the 0–10 soil profile and from each experimental subunit 10 subsamples were taken. Each sample contained 200 g soil or growing medium and was divided into homogenous subsamples: one subsample was used for chemical analyses (100 g) and water content (50 g), one subsample was immediately after sampling stored on dry ice, preserved at −80°C and used for molecular microbial community analysis (50 g). The ammonium and the nitrogen content, the pH and the electrical conductivity (EC) in the 0–10 cm organic soil layer and in the growing medium were taken at the start 55 DAS(T0), 68 DAS (T1), 83 DAS (T2), 113 DAS (T3), 146 DAS (T4), 172 DAS (T5), 221 DAS (T6), and 321 DAS (T7). Samples for microbial community analysis were taken at 8 different timepoints, i.e., 55 DAS, 68 DAS, 83 DAS, 113 DAS, 146 DAS, 172 DAS, 221 DAS, and 321 DAS. Samples for PLFA analyses were collected at four different time points, i.e., 55 DAS, 83 DAS, 221 DAS, and 321 DAS. Briefly, for the PLFA analyses the soil and growing media were freeze-dried using a modified technique (Bligh and Dyer, 1959). Whole plants were harvested, chopped and samples from stem and leaves without tomatoes were collected for analysis at 221 DAS and 321 DAS.

### Nitrogen Determination in the Soil, Soilless Culture System and Plant

Physicochemical characteristics of the soil and soilless culture systems were determined at the start and throughout the whole experimental period. Potassium, phosphorus, calcium, magnesium, iron and manganese were extracted in ammonium acetate and measured with ICP. The electrical conductivity (EC), pH(H_2_O), ammonium (NH_4_^+^), nitrate (NO_3_^–^), sulfate (SO_4_^2–^) and sodium (Na^+^) were measured in a water extract according to EN 13038, EN 13037 and EN 13652, respectively. Nitrate was measured with an Dionex DX-3000 IC ion chromatograph (Dionex, Sunnyvale, CA, United States). Ammonium was measured by steam distillation (Bremner and Keeney, 1965). The elements were measured by a ICP-OES (VISTA-PRO, Varian, Palo Alto, CA, United States). The total nitrogen content of the plants sampled was determined according to Dumas (British Standards Institute Staff, 2001).

### Estimation of the Nitrogen Dynamics in Soil and Soilless Culture Systems

The ammonium and the nitrogen concentration in the soil at the start 55 DAS – time point 1, 221 DAS – time point 2 and 321 DAS – time point 3) were calculated based on the ammonium, the nitrate and the estimated soil dry bulk density (1.25 kg L^–1^) of the 0–10 cm soil layer. Fertilizers were applied in the top layer of the organic soil (0–10 cm) and soil water content was controlled in the 0–10 cm layer. The ammonium and the nitrogen concentration of GBOF and GBFISH were also determined and were recalculated based on the amount of growing medium needed per ha, i.e., 90 m^3^ ha^–1^. At time point 2 and 3 the dry matter and N content of whole plant samples were determined for calculation of dry biomass and total N uptake at time point 2 and 3. Samples were taken from shredded tomato plants (*n* = 4). Samples were dehydrated in an oven at 70°C for 48 h. N content was determined on chopped dehydrated plant material (Kjeldahl method, ISO 5983-2).

### Plant Performance

The length of the plant was measured on a weekly basis with a measuring tape. Both the fresh and dry weight of the plants and nitrogen content were determined at the start (55 DAS), the middle 221 DAS and at the end 321 DAS of the experiment. Tomatoes were harvested on a weekly basis or whenever necessary and cumulative yield (fresh weight) was determined.

### DNA Extraction

Total DNA was extracted using physical disruption with the bead beating method from Hernandez-Sanabria et al. (2020) and Grunert et al. (2016a). Cells were lysed in a FastPrep-96 homogenizer (MP Biomedicals, Illkirch, France) and DNA was precipitated with cold ethanol and resuspended in 30 μl of TE buffer (10 mM Tris-HCl, 1 mM EDTA [pH 8.0]). Concentration and quality of DNA were measured based on the absorbance at 260 and 280 nm in a Nanodrop ND 1000 spectrophotometer (NanoDrop Technologies, Wilmington, DE, United States).

### Total Biomass and Overall Fingerprint of the Viable Fungal and Bacterial Communities

Microbial community composition was determined by phospholipid fatty acid analysis (PLFA). Briefly, PLFAs were obtained from freeze-dried soil and growing media using a modified technique (Bligh and Dyer, 1959) The PLFAs were determined using a procedure modified from Balser (2001) and Moeskops et al. (2010). To identify Gram-positive bacteria, the sum of i14:0, i15:0, a15:0, i16:0, a16:0, i17:0, and a17:0 was computed. The fatty acids cy17:0, cy17:0new, cy19:0 and cy19:0 new were considered to be representative for Gram-negative bacteria. The sum of 10Me16:0, 10Me17:0 and 10Me18:0 were an indicator for the Actinomycetes. The fatty acid 18:2ω6c was used as fatty acid for fungi, and two alternative signature fatty acids for fungi were considered as well, i.e., 18:1ω9 and 18:3 ω3. The fatty acid 16:1ω5c was an indicator for AMF. Bacteria: fungi (B:F) ratios were calculated by dividing the sum of markers for Gram-positive, Gram-negative bacteria, 15:0 and 17:0 by the fungal marker 18:2ω6c.

### Bacterial Community Structure and Composition

Total DNA was extracted from the growing medium samples using the Power Soil^®^ DNA Isolation Kit (MoBio Laboratories Inc., Carlsbad, CA, United States). Five hundred milligrams were used from the bulk as previously described (Grunert et al., 2019). High-throughput amplicon sequencing of the V3 – V4 hypervariable region (Klindworth et al., 2012) was performed with the Illumina MiSeq platform (LGC Genomics GmbH, Berlin, Germany) and the following primers were used 338f and the 518 r (Øvreås et al., 1997). Bioinformatics and data pre-processing followed a protocol developed in-house (El Hage et al., 2019; Grunert et al., 2019; Hernandez-Sanabria et al., 2020). A generalized linear mixed model was employed to compute the effect of tomato cultivating system and time and the interactions between “tomato cultivating system” and time on each individual genus (El Hage et al., 2019; Grunert et al., 2019; Hernandez-Sanabria et al., 2020). Differences among library size sample were accounted for with the offset option in proc GLIMMIX in SAS (Paschold et al., 2012; El Hage et al., 2019). P values for each comparison were converted to q-values that were then used to identify differences in relative abundances of bacterial genera while controlling the false discovery rate (FDR) at the 5% level (Storey, 2015).

### Evaluating Relationships Between Microbial Community Characteristics and Culturing System Features

Variations in tomato cultivation systems and nitrogen dynamics were computed using a repeated measures mixed in SAS (version 9.4, SAS Institute, Cary, United States). *P*-values for Pearson correlation coefficients and regression coefficients were used to determine significant relations with a significance level of *P* < 0.05 (Grunert et al., 2019). Multiple Factor Analysis (MFA) was employed to detect how the microbial community composition based on a PLFA analysis and bacterial abundance contributed to the differences between the four different tomato cultivating systems across time points. In addition, MFA was applied to assess the correlations between the chemical and microbiological variables based on the PLFA analysis and bacterial abundance detected in the four tomato cultivating systems. R was used (Grunert et al., 2019; Hernandez-Sanabria et al., 2020) to compute the function MFA from the FactoMineR package (Lê et al., 2008).

Richness, Fishers diversity, Shannon, Simpson and inverse Simpson indices were used to calculate the alpha diversity within each sample. Pielou was used as an index of evenness in the community. Variations in alpha diversity and evenness indexes between treatments were statistically analyzed using a repeated measures mixed model in SAS (version 9.4, SAS Institute, Cary, United States) with the four tomato cultivating systems as a fixed effect and time. This method allowed us to attribute the differences in the diversity measures to time or tomato cultivating system or to the interaction of the two factors.

Chao and Bray–Curtis indices were used to check dissimilarity and find out the impact of experimental factors on microbial community composition. Principal Coordinate Analysis (PCoA) visualized the differences between samples, using the vegan package in R (Oksanen et al., 2007), and stratified permutational multivariate analysis of variance (PERMANOVA) with 999 permutations were run to display the significance of each covariate on the microbial community of the bulk soil (Grunert et al., 2019). ANOVA was applied to reveal whether the distribution of the genera was different between treatments (Oksanen et al., 2007).

### Statistical Analysis

The data about the fresh plant weight, cumulative yield, the amount of red tomatoes, the percentage of green tomatoes and percentage of blossom end rot were not statistically analyzed. The length of the plant was measured on a weekly basis for the 5 experimental units and for the 5 plants per experimental unit with a measuring tape and a 95% confidence interval was plotted for the different treatments for the first 120 days (55 DAS till 175 DAS).

## Results

### Similar Plant Performance Was Observed in Soilless Culture Systems and Soil Based System and Yield Was the Highest With the Aquaponics-Derived Fertilizer

**FIGURE 2:**
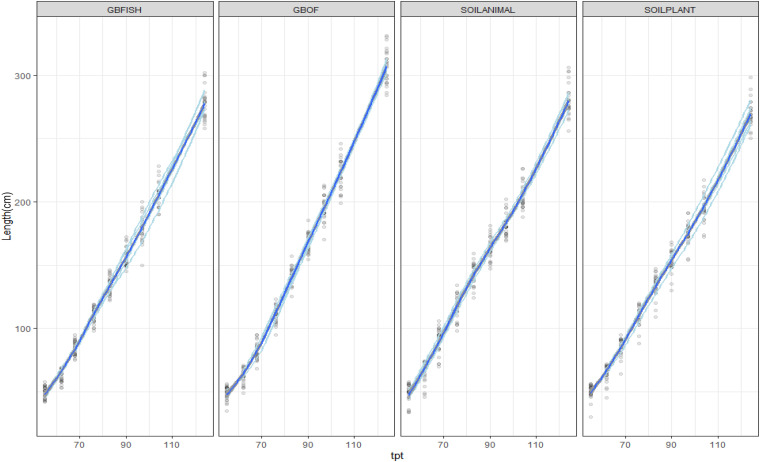
Evolution of the plant length (cm) from 55 DAS till 125 DAS in the growing medium. i.e., GBOF and GBFISH and the organic soil. i.e., SOILANIMAL and SOILPLANT. The graph shows the 95% confidence interval, which was plotted around the dark blue line. The light blue lines are the values of the different experimental units.

Plant length was followed during the whole experimental period (55 DAS till 321 DAS). Figure 2 shows the evolution of the plants length from 55 DAS till 175 DAS. The final plant length for GBOF, GBFISH, SOILANIMAL, and SOILPLANT was 6.96 ± 0.06 m, 6.74 ± 0.07 m, 7.04 ± 0.03 m, and 7.04 ± 0.04 m, respectively. The average fresh plant weight follows a similar trend as the plant length (Figure 2). The average fresh plant weight showed significant differences (*P* < 0.05) for GBOF><0.05) for GBOF, GBFISH, SOILANIMAL and SOILPLANT was 4.081^*a*^ ± 0.901 kg.m^–2^, 3.074 ± 0.557^*b*^ kg.m^–2^, 4.081 ± 1.034^*a*^ kg.m^–2^ and 4.691 ± 0.477^*a*^ kg.m^–2^, respectively. The dry matter content of the plants was 0.481 ± 0.051 kg.m^–2^, 0.31 ± 0.054 kg.m^–2^, 0.44 ± 0.080 kg.m^–2^, and 0.49 ± 0.089 kg.m^–2^, respectively. Table 1 shows that the cumulative yield of the system with soilless organic growing medium plus inorganic fertilizer (GBFISH) resulted in higher tomato yield (kg of tomatoes) in comparison with the three other cultivation systems. After 117 days since plantation, the cumulative yield dropped and followed the same trend as that observed in the soil supplied with either fertilizer.

### Fast-Paced Fluctuations in pH(H_2_O) and Nutrient Cycling Processes Were Observed in Growing Medium

**FIGURE 3:**
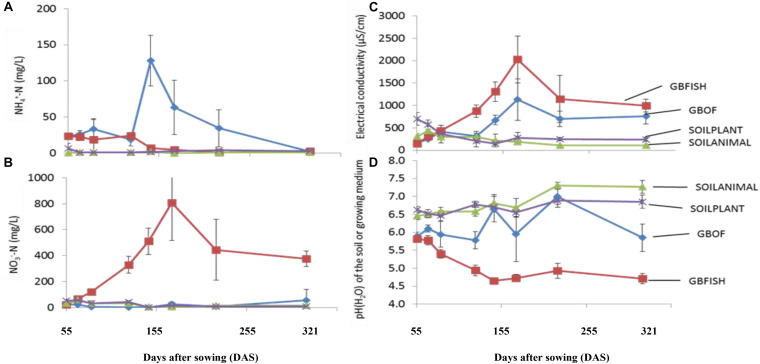
Evolution of the ammonium concentration **(A)**. Nitrate concentration **(B)**. The electrical conductivity **(C)** and pH(H_2_O) **(D)** in the growing medium. i.e., GBOF and GBFISH and the organic soil. i.e., SOILANIMAL and SOILPLANT during the whole experimental period which lasted 321 days. The ammonium and the nitrogen content, the pH and the electrical conductivity (EC) in the 0–10 cm organic soil layer and in the growing medium were taken at the start 55 DAS (T0), 68 DAS (T1), 83 DAS (T2), 113 DAS (T3), 146 DAS (T4), 172 DAS (T5), 221 DAS (T6), and 321 DAS (T7).

Evolution of the electrical conductivity, pH(H_2_O) and nitrate and ammonium concentration (Figure 3) was followed over time. Electrical conductivity, nitrate, ammonium, phosphorus, potassium, sodium and chloride differed among the four different tomato cultivating systems (*P* < 0.001) and time was a factor significantly influencing these traits (*P* < 0.001, Table 2). Soil fertilized with animal-derived material (243 ± 111 μS.cm^–1^) or plant-derived material (344 ± 192 μS.cm^–1^) showed the lowest average electrical conductivity, which decreased over time. On the contrary, the soilless culture system showed increasingly higher values (Figure 3) (GBOF = 551 ± 323 μS cm^–1^ and GBFISH = 905 ± 614 μS cm^–1^). The pH(H_2_O) of the soil increased over time for SOILANIMAL from 6.3 to 7.3 and SOILPLANT from 6.6 to 6.9, while the pH(H_2_O) in the organic growing medium was very dynamic and fluctuated over time (Figure 3). The pH(H_2_O) of GBFISH dropped between days 13 and 91 from 5.7 to 4.6 indicating increased uptake of cations, such as potassium and ammonium. Indeed, we found an increased amount of ammonium in GBFISH until days 69. GBOF, however, showed the highest ammonium concentration (41.2 ± 39.1 mg NH_4_^+^-N L^–1^), while nitrate was significantly higher for GBFISH (*P* < 0.05, Figure 3) and decreased over time in soil supplemented with either fertilizer.

**TABLE 2:** Average values of 11 different variables throughout the whole experimental period with standard error.

Variable	Treatment (trt)				*p*-value (trt)	*p*-value (time)
	GBOF	GBFISH	SOILANIMAL	SOILPLANT		
pH(H_2_O)	6.1 ± 0.04^b^	5.1 ± 0.04^a^	6.8 ± 0.05^c^	6.6 ± 0.05^c^	<0.001	<0.001
EC (μS cm^–1^)	596 ± 34^c^	883 ± 34^d^	243 ± 37^a^	364 ± 40^b^	<0.001	<0.001
NO_3_^–^-N (mg L^–1^*)	14 ± 12^a^	332 ± 12^c^	23 ± 13^b^	25 ± 14^b^	<0.001	<0.001
NH_4_^+^-N (mg L^–1^)	40 ± 2^c^	12 ± 2^b^	1 ± 2^a^	3 ± 2^a^	<0.001	<0.001
NO_3_^–^-N/NH_4_^+^-N ratio	0.35	27	23	8		
P (mg L^–1^)	218 ± 14^b^	216 ± 14^b^	37 ± 15^a^	35 ± 16^a^	<0.001	<0.001
K^+^ (mg L^–1^)	382 ± 12^c^	423 ± 12^d^	58 ± 13^a^	88 ± 15^b^	<0.001	<0.001
Ca^2+^ (mg L^–1^)	1234 ± 33^b^	1173 ± 34^b^	937 ± 36^a^	1103 ± 38^b^	<0.001	<0.001
Mg^2+^ (mg L^–1^)	254 ± 9^c^	286 ± 9^d^	138 ± 10^a^	182 ± 10^b^	<0.001	<0.001
SO_4_^2–^ (mg L^–1^)	740 ± 40^c^	708 ± 40^b^	448 ± 44^a^	799 ± 4^c^	<0.001	<0.001
Na^+^ (mg L^–1^)	118 ± 4^c^	97 ± 6^b^	33 ± 7^a^	42 ± 7^a^	<0.001	<0.001
Cl^–^ (mg L^–1^)	340 ± 20^c^	43 ± 20^b^	18 ± 22^a^	18 ± 24^a^	<0.001	<0.001

*n = 5. Differences in variables among treatments were compared using a repeated measures mixed model in SAS. For each column, different superscripts indicate significantly different means according to Duncan’s multiple range test (*P* < 0.05).*

### Physicochemical Characteristics Changed Over Time and Interacted With Bacterial Community Metrics

**FIGURE 4:**
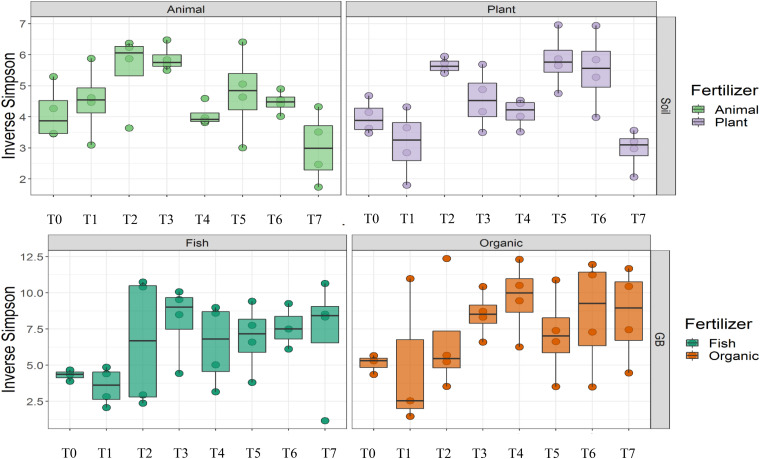
Culture system impacted bacterial community characteristics and showed opposite trends. Alpha diversity was lower and oscillated over time in soil **(upper panel)**, while it steadily increased in soilless systems and remained high at the end of the experiment **(lower panel)**. Samples for microbial community analysis were taken at 8 different timepoints, i.e., 55 DAS (T0), 68 DAS (T1), 83 DAS (T2), 113 DAS (T3), 146 DAS (T4), 172 DAS (T5), 221 DAS (T6), and 321 DAS (T7).

**FIGURE 5:**
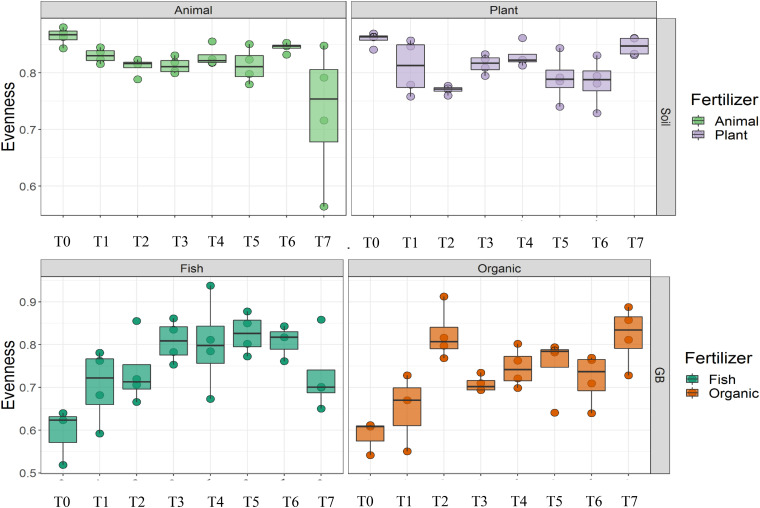
Culture system impacted bacterial community characteristics and showed opposite trends. On the contrary, evenness was consistently higher in soil **(upper panel)**, while it tend to decrease in the aquaponics soilless system. **(lower panel left)** Samples for microbial community analysis were taken at 8 different timepoints, i.e., 55 DAS (T0), 68 DAS (T1), 83 DAS (T2), 113 DAS (T3), 146 DAS (T4), 172 DAS (T5), 221 DAS (T6), and 321 DAS (T7).

**FIGURE 6:**
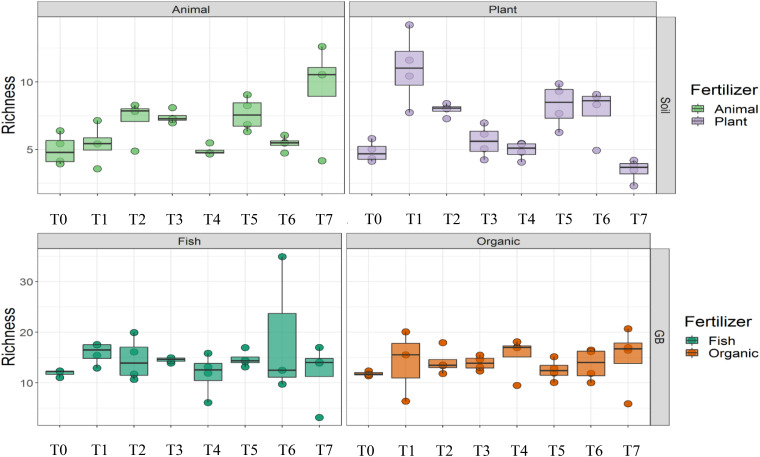
Culture system impacted bacterial community characteristics and showed opposite trends. Richness followed the oscillatory trend in soil **(upper panel)** but not in growing medium **(lower panel)**. Samples for microbial community analysis were taken at 8 different timepoints, i.e., 55 DAS (T0), 68 DAS (T1), 83 DAS (T2), 113 DAS (T3), 146 DAS (T4), 172 DAS (T5), 221 DAS (T6), and 321 DAS (T7).

Cultivation system (*P* < 0.01), time (*P* < 0.01) and the interaction between tomato cultivation system and time (*P* < 0.01) significantly influenced species richness, diversity and evenness (Pielou’s index) (Table 3). Alpha diversity (Figure 4) oscillated in the soil, while remaining consistent on the soilless system throughout time. On the contrary, evenness (Figure 5) was persistently high in soil but not in growing medium and significantly decreased at the final harvest in soilless culture supplemented with fish fertilizer (GBFISH). Richness (Figure 6) followed the same trend observed for alpha diversity and shifted toward a decrease with plant-derived fertilizer but not when blood meal was added. These results indicate that soil is a highly dynamic environment, where bacterial communities are rapidly adapting, while community characteristics of bacteria inhabiting growing medium stay uniform over time despite fertilizer application.

**TABLE 3:** Effect of tomato cultivation system (GBOF, GBFISH, SOILANIMAL, and SOILPLANT) on species richness (total species), diversity (Shannon, Fisher’s alpha, Simpson and Inverse Simpson indices), and evenness (Pielou’s index) for all the 8 time points (*n* = 3).

Index	Time	Tomato cultivation system (treatment)				SEM	Effect		
		GBOF	GBFISH	SOILANIMAL	SOILPLANT		Treatment	Time	Treatment* time interaction
Total species	0	124.3^a^	98.8^a^	332.8^b^	336.8^b^	37.3	<0.0001	<0.0001	<0.0001
	1	84.5^ab^	52.8^a^	413.5^c^	116.0^b^				
	2	100.5^a^	143.0^b^	394.3^c^	437.3^d^				
	3	295.0^a^	205.3^b^	412.0^c^	413.5^c^				
	4	281.5^a^	203.5^b^	406.5^c^	403.3^c^				
	5	265.5^b^	105.5^a^	321.5^c^	382.3^d^				
	6	339.5^b^	145.3^a^	385.8^c^	403.0^c^				
	7	179.8^a^	223.3^b^	151.0^a^	409.0^c^				
Pielou	0	0.625^a^	0.595^a^	0.783^b^	0.793^b^	0.024	<0.0001	<0.0001	<0.0001
	1	0.647^a^	0.706^a^	0.777^b^	0.792^b^				
	2	0.810^a^	0.713^c^	0.772^b^	0.760^b^				
	3	0.708^a^	0.758^a^	0.784^b^	0.775^b^				
	4	0.750	0.760	0.768	0.772				
	5	0.753	0.795	0.785	0.781				
	6	0.736	0.726	0.785	0.776				
	7	0.714^a^	0.687^b^	0.700^c^	0.767^a^				
Shannon	0	2.778^a^	2.563^a^	4.544^b^	4.609^b^	0.211	<0.0001	<0.0001	<0.0001
	1	2.709^a^	2.794^a^	4.678^b^	3.764^c^				
	2	3.568^a^	3.293^a^	4.616^b^	4.618^b^				
	3	4.022^a^	3.952^a^	4.718^b^	4.665^b^				
	4	4.211^a^	3.912^a^	4.613^b^	4.626^b^				
	5	4.062^a^	3.631^a^	4.484^b^	4.640^b^				
	6	4.288^a^	3.256^b^	4.672^c^	4.651^c^				
	7	3.602^a^	3.647^a^	3.302^b^	4.613^c^				
Simpson	0	0.862^a^	0.812^a^	0.973	0.977	0.028	<0.0001	<0.0001	<0.0001
	1	0.790^a^	0.816^a^	0.979	0.921				
	2	0.935^a^	0.891^a^	0.978^b^	0.979^b^				
	3	0.964	0.960	0.982	0.980				
	4	0.969	0.928	0.976	0.976				
	5	0.955	0.936	0.974	0.980				
	6	0.963^a^	0.858^b^	0.979^c^	0.979^c^				
	7	0.934	0.899	0.858	0.974				
Inverse Simpson	0	7.427^a^	6.049^a^	38.127^b^	44.223^c^	4.884	<0.0001	<0.0001	<0.0001
	1	7.891^a^	6.564^a^	48.304^b^	14.460^c^				
	2	19.618^a^	13.168^a^	46.218^b^	47.679^b^				
	3	27.817^a^	26.136^a^	55.706^b^	49.539^c^				
	4	33.674^a^	32.469^a^	41.571^b^	42.066^b^				
	5	33.669^a^	21.518^a^	41.184^b^	50.692^c^				
	6	37.159^a^	18.664^b^	47.643^b^	48.561^c^				
	7	16.600^a^	19.257^a^	15.655^b^	38.384^c^				
Fisher’s	0	26.966^a^	22.582^a^	63.810^b^	63.979^b^	4.640	<0.0001	<0.0001	<0.0001
	1	23.416^a^	22.138^a^	68.101^b^	51.262^c^				
	2	34.273^a^	35.035^a^	65.298^b^	69.475^b^				
	3	47.982^a^	48.614^a^	68.776^b^	66.327^b^				
	4	48.196^a^	49.268^a^	67.699^b^	66.150^b^				
	5	50.323^a^	37.039^b^	62.389^c^	67.027^c^				
	6	55.162^a^	36.092^b^	69.952^b^	68.985^b^				
	7	37.651^a^	45.136^a^	35.967^b^	67.726^c^				

*GBOF, organic growing medium with organic fertilizer; GBFISH, organic growing medium with fish effluent; SOILANIMAL, organic soil with animal derived material as fertilizer (blood meal); and SOILPLANT, organic soil with plant derived material as fertilizer (malt sprouts). NS, not significant effect. For each column, different superscripts indicate significantly different means according to Duncan’s multiple range test (*P* < 0.05). SEM, standard error of the mean.*

**FIGURE 7:**
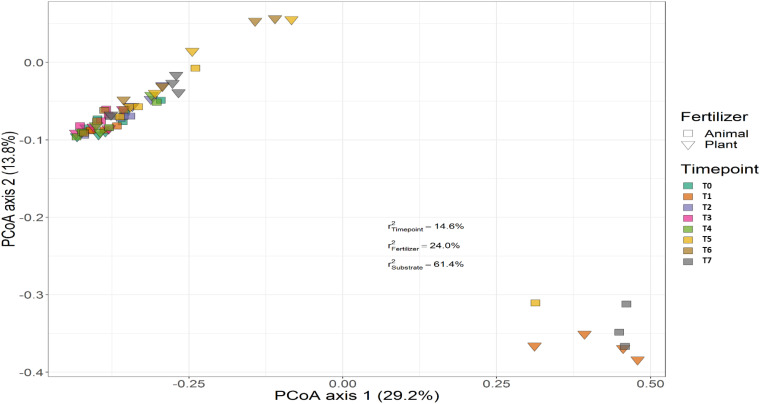
Beta diversity of bacterial communities in soil harboring tomato plants and supplemented with different fertilizers. Squares indicate communities from soil fertilized with animal-origin fertilizer and inverted triangles show communities supplemented with plant-derived fertilizer. Samples of soil were followed over time to observe the bacterial community development. A color code on the right indicates the community sampled at different time points (time point 0-time point 8). “Substrate” indicates whether culture system contributed to the variance among bacterial communities. PERMANOVA results indicate that substrate (soil or growing medium) is the factor explaining the highest percentage of the variance among communities in the rhizosphere in both culture systems.

**FIGURE 8:**
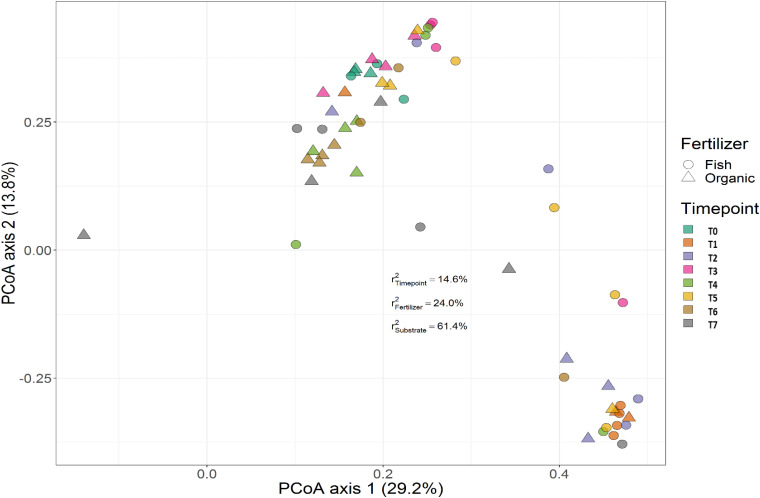
Beta diversity of rhizosphere bacterial communities in growing media harboring tomato plants and supplemented with different fertilizers. Circles indicate communities supplemented with GBFISH. and triangles show bacterial communities amended with organic fertilizer (GBOF). Samples of growing media were followed over time to observe the bacterial community development. A color code on the right indicates the community sampled at different time points (time point 0-time point 8). “Substrate” indicates whether culture system contributed to the variance among bacterial communities. PERMANOVA results indicate that communities inhabiting growing medium were significantly different from those in soil, even if they hosted the same cultivar.

Treatment (*P* < 0.001), time (*P* < 0.001) and the interaction between treatment and time (*P* < 0.001) had a significant effect on the relative abundances of the bacterial genera. PERMANOVA showed that communities in soil remained similar throughout the trial (*P* < 0.001), while those in the growing medium were significantly different at the beginning and converged over time. Figures 7, 8 show the beta diversity of bacterial communities in the soil harboring tomato plants and supplemented with different fertilizers. “Substrate” indicates whether the culture system contributed to the variance among bacterial communities. PERMANOVA results indicate that substrate (soil or growing medium) is the factor explaining the highest percentage of the variance among communities in the rhizosphere in both culture systems and PERMANOVA results indicate that communities inhabiting growing medium were significantly different from those in soil, even if they hosted the same cultivar.

**FIGURE 9:**
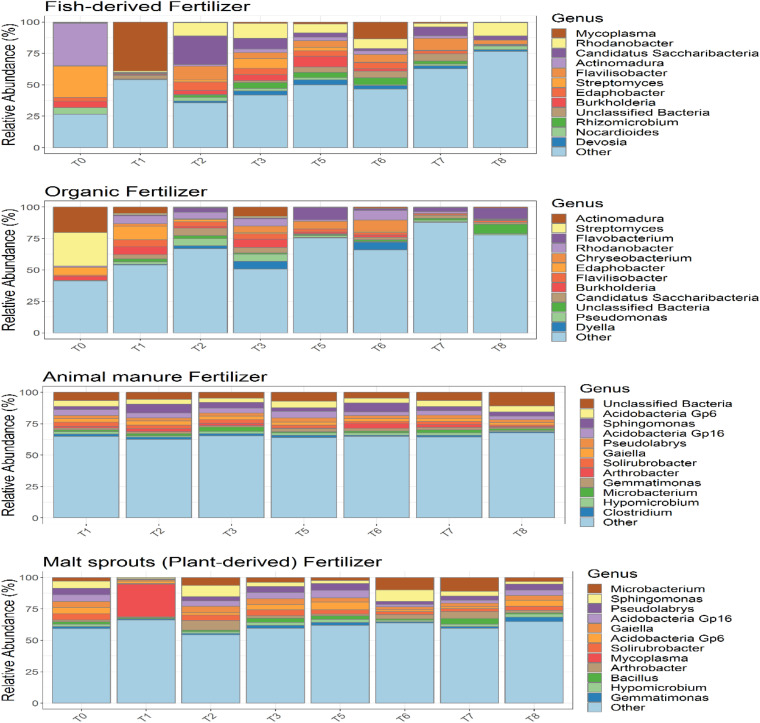
Analysis of the bacterial composition of the different tomato cultivation systems: GBOF (organic fertilizer), GBFISH (fish derived fertilizer), SOILANIMAL (animal based fertilizer) and SOILPLANT (plant derived fertilizer). Relative abundance (percentages) of the different bacterial phyla (16S V3–V4 region) in the tomato bulk soil. Phyla representing less than 1% of the total community are bundled in the group “other”, as their taxonomic composition may be uncertain.

Community composition on soil plus either fertilizer remained unaltered over time (Figure 9A), excepting on the first time point of soil supplemented with plant-derived fertilizer, when the relative abundance of *Mycoplasma* was significantly increased. Unclassified bacteria and *Clostridium* were taxa exclusively present when soil was amended with animal manure, while the relative abundance of *Bacillus* increased when plant-derived fertilizer was added. On the contrary, community composition of soilless systems (Figure 9B) was impacted by fertilizer and *Mycoplasma*, *Rhizomicrobium*, *Nocardioides* and *Devosia* were only detected in the aquaponics, while *Pseudomonas*, *Dyella*, and *Flavobacterium* increased relative abundance in soilless systems with organic fertilizer. Thus, differences in the rhizosphere bacterial community composition were mainly impacted by fertilizer within soilless systems, while the opposite occurred in soil.

**FIGURE 10:**
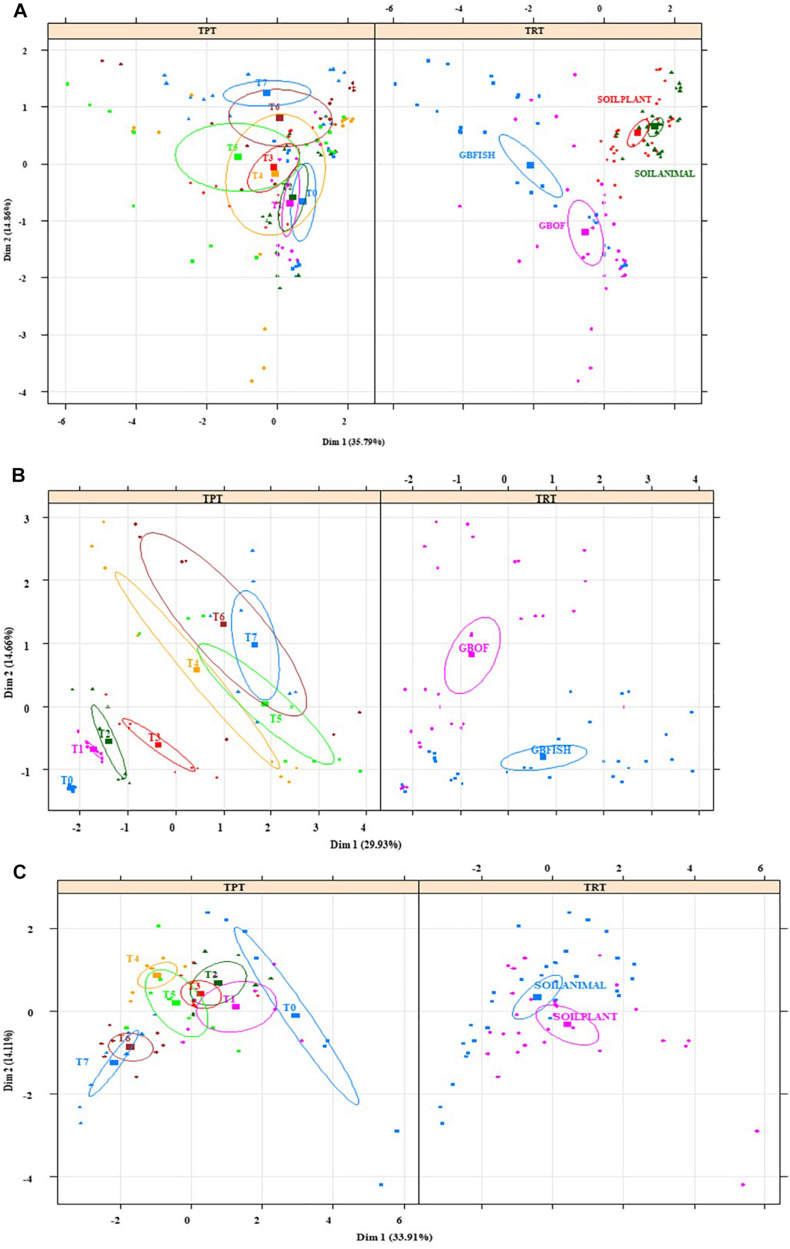
Multiple factor analysis in which 20 of the most significant factors were taken into account for the four contrasting culture systems **(A)**, organic soil **(B),** and for the soilless culture system **(C)**. TRT indicates the tomato cultivating system (GBOF, organic growing medium with organic fertilizers; GBFISH, organic growing medium with fish effluent; SOILANIMAL, organic soil that was fertilizer with animal-derived material; and SOILPLANT, soil that was fertilized with plant-derived material). TRT, time point. Samples for microbial community analysis were taken at 8 different timepoints, i.e., 55 DAS (T0), 68 DAS (T1), 83 DAS (T2), 113 DAS (T3), 146 DAS (T4), 172 DAS (T5), 221 DAS (T6), and 321 DAS (T7). Circles indicate the 95% confidence interval.

Multiple Factor Analysis showed that the environment in the soil (Figures 10A–C) at the start of the cropping (T0) was significantly different and the variance (given by the size of the confidence ellipse) reduced over time. The opposite happened in the soilless culture system (Figures 10A–C) and bacterial relative abundances were similar at the beginning, but variance increased over time and GBOF and GBFISH become differentiated as a result of the fertilizer used. Multiple factor analyses (Figures 10A–C) of the soil showed that plant length, pH, *Flavisolibacter*, phosphorus, chloride, ammonium, potassium, calcium, magnesium, sodium, electrical conductivity, nitrate, sulfate, *Desulfotomaculum*, *Solirubrobacter*, *Dehalococcoides*, *Bythopirellula*, *Steroidobacter*, *Litorilinea*, *Nonomuraea* were the variables significantly discriminating between SOILANIMAL and SOILPLANT. The first dimension (33.9% of variance) of the soil was positively correlated with T0 (*P* < 0.001), and T1 (*P* < 0.05) and negatively correlated with T6 (*P* = 0.006) and T7 (*P* = 0.0003), whereas the second dimension (14.1% of variance) was positively correlated with SOILANIMAL (*P* < 0.03), and T4 (*P* = 0.03) and negatively correlated with T6 (*P* = 0.03), SOILPLANT (*P* = 0.003) and T7 (*P* = 0.001). In contrast, nitrate, *Acidobacteria Gp 14*, *Rhizomicrobium*, *Unclassified bacteria*, *Verrucomicrobia SD3*, magnesium, electrical conductivity, *Parcubacteria*, sulfate, sodium, potassium, phosphorus, plant length, calcium, chloride, *Amaricoccus*, *Gemmobacter*, ammonium, *Brevundimonas*, pH were the variables discriminating between GBOF and GBFISH (Figures 10A–C). The first dimension (29.9% of variance) of the soilless culture system was positively correlated with GBFISH (*P* = 0.001), T5 (*P* = 0.001), T7 (*P* = 0.005) and negatively correlated with T2 (*P* = 0.02), T1 (*P* = 0.001), T0 (*P* = 0.002) and GBOF (*P* < 0.001), whereas the second dimension (14.7% of variance) was positively correlated with GBOF (*P* < 0.001), T6 (*P* = 0.004), and T7 (*P* = 0.002) and negatively correlated with T0 (*P* = 0.009) and GBFISH (*P* < 0.001).

### Long-Term Fertilizer Regimes Significantly Changed the PLFA Fingerprints in Both the Soilless Culture and Soil Based Culture System

**FIGURE 11:**
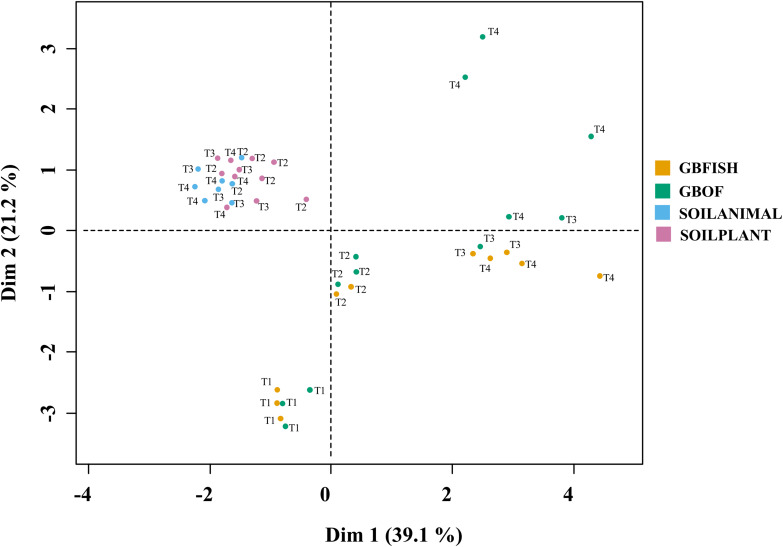
Multiple factor analysis in which 11 chemical soil and growing medium characteristics [pH(H_2_O)]. EC (nitrate, ammonium, phosphorus, potassium, calcium, magnesium, sulfate, sodium, and chloride) and eight microbial characteristics [Gram-positive, Gram-negative bacteria, fungi 18:1, fungi 18:2, fungi 18:3, Actinomycetes, arbuscular mycorrhizal fungi (AMF), and protozoa] were used to discriminate between the different tomato cultivation systems. GBOF, organic growing medium with organic fertilizers. GBFISH, organic growing medium with fish effluent. SOILANIMAL, organic soil that was fertilizer with animal-derived material; and SOILPLANT, soil that was fertilized with plant-derived material. Samples for PLFA analyses were collected at four different time points, i.e. 55 DAS (T1), 83 DAS (T2), 221 DAS (T6) and 321 DAS (T7).

Combinations of organic soil with plant and animal-derived material and organic growing medium with fish effluent and organic fertilizer differed in its characteristics throughout the experimental period. Eleven soil and growing media characteristics and eight microbial characteristics (gram-positive, gram-negative bacteria, 18:1, 18:2, and 18:3 fungi, actinomycetes, arbuscular mycorrhizal fungi and protozoa) were analyzed together in a MFA (Figure 11). Overall, long-term fertilizer regimes significantly alternated the PLFA marks in both the soilless culture and soil based culture system.

Regarding to the four different tomato cultivation systems, the first two dimensions (Supplementary Table S5) accounted for 60.3% of the total variance, indicating a good reproducibility of the data of the 5 experimental units per treatment. The first dimension is positively correlated with GBOF (*P* < 0.05) and GBFISH (*P* < 0.05) and negatively correlated with SOILANIMAL and SOILPLANT (Supplementary Table S5), whereas the second dimension 2 is positively correlated with SOILPLANT (*P* < 0.05) and time point 4 (*P* = 0.014) and negatively correlated with GBFISH and timepoint 1. Dimension 3 is positively correlated with GBOF (*P* < 0.001) and negatively correlated with GBFISH. Dimension 4 is negatively correlated with SOILANIMAL (*P* < 0.05).

The soilless culture systems GBOF and GBFISH, showed a positive correlation (dimension 1) with potassium, protozoa, electrical conductivity, the fungal FAME marker 18:3, sodium, total biomass, calcium, phosphorous, nitrate, fungal FAME marker 18:2, chloride, magnesium and sulfate, while the soil based system is positively correlated with actinomycetes, pH, the bacteria to fungi ratio and the gram-positive bacteria. GBOF showed a positive correlation (dimension 3) with ammonium, the total biomass, chloride, pH, magnesium and phosphorous, while GBFISH was positively correlated with fungal FAME marker 18:2 and fungal FAME marker 18:3 and nitrate. SOILPLANT, SOILANIMAL and timepoint 4 were positively correlated with AMF (arbuscular mycorrhizal fungi), gram-negative bacteria, the bacteria to fungi ratio, calcium, sulfate, phosphorous, gram-positive bacteria, pH, sodium and chloride, while GBFISH was correlated with the fungal FAME marker 18:3, ammonium, actinomycetes, the fungal FAME marker 18:2 and the fungal FAME marker 18:1.

## Discussion

Healthy soils are decisive to biodiversity and play a paramount role in fighting climate change. Soils are a non-renewable resource on which 95% of our food supply depends. Organic agriculture is a production system that nurses the health of soils, ecosystems and people. It builds on ecological processes, biodiversity, instead of using resources with inimical effects. Soilless culture systems and consequently growing media are also an fundamental sector of the food supply chain. Growing media facilitates sustainable horticulture and protected culture systems are vital for producing fruits and vegetables. Both (organic) soil based and soilless culture systems are principal members in the transition toward a more sustainable food production. It has been shown that it is possible to grow tomatoes in soilless culture systems in combination with inorganic and organic fertilizers (Heeb et al., 2005b; Zhai et al., 2009; Gravel et al., 2010a; Grunert et al., 2016a), in aquaponic systems (Suhl et al., 2016) and in organic soil based systems (Bélair and Tremblay, 1995; Gravel et al., 2010b). Moreover, it was demonstrated that soil and soilless culture systems and soil amendments and fertilizers can have a clear impact on plant growth, tomato fruit quality and on the suppression of plant diseases (Cotxarrera et al., 2002; Gruda, 2008; Zhai et al., 2009; Coppens et al., 2015; Sakarika et al., 2019; Dion et al., 2020). Several research papers have associated these favorable effects on the microbiome of the soil and the rhizosphere of the plant (Tu et al., 2006; El-Yazeid and Abou-Aly, 2011; Bona et al., 2018; Sellitto et al., 2019). In addition, it has been proven that temporal, biotic and abiotic components have considerable influence on the bulk microbiome and the rhizosphere of tomatoes (Maloney et al., 1997; Grunert et al., 2016a; Grunert et al., 2019). In the presented study, we cultivated tomato plants grown in soil based and soilless culture systems. We hypothesized that (i) the soil and soilless culture based edaphic properties, which are strongly altered during one tomato growing season, consecutively affect the bacterial and fungal microbial structure. Moreover, we hypothesized that (ii) the community changes caused by four contrasting fertilization strategies included shifts in the abundance of various plant-beneficial soil- and soilless culture based microorganisms, thus influencing plant performance.

A total of 11 chemical variables were monitored for the four different tomato cultivating systems. No major plant growth anomalies were found, except for GBOF with the highest nitrogen supply rate. We found a significant effect tomato cultivating system and time on species richness. Our MFA analysis based on the PLFA results (Figure 11) and based on high throughput sequencing of the 16S rRNA gene and chemical factors, indicate that the dispersion of the genera among treatments is significantly different. Long-term fertilizer regimes significantly changed the PLFA fingerprints in both the soilless culture and soil based culture system. Indeed, the diversity of microbial communities associated with the soil or soilless culture system are directly influenced by the physical and chemical properties of the soil. It must be considered that four different tomato cultivating systems supplemented with different fertilizers were compared with each other, making it impossible to estimate the separate effect of soil type or growing medium or fertilizer used on the microbial community composition. However, SOILANIMAL and SOILPLANT displayed similar microbial community composition, i.e., richness, evenness and GBOF and GBFISH also revealed a more similar microbial community composition indicating a potential soil or growing medium effect. On the other hand, within the organic soil and soilless culture system the microbial community composition seemed to be different depending on the fertilizer used (animal or plant based nutrients or organic versus inorganic). Alpha diversity (Figure 4) oscillated in the soil, while remaining consistent in the soilless system throughout time. On the contrary, evenness (Figure 6) was persistently high in soil but not in growing medium and significantly decreased at the final harvest in soilless culture supplemented with fish fertilizer (GBFISH). Richness (Figure 5) followed the same trend observed for alpha diversity (Figure 4) and shifted toward a decrease with plant-derived fertilizer but not when blood meal was added. In many cases, alterations in evenness crop up with little or no changes in species richness, and this illustrates the pertinence of evenness as a component of diversity (Wilsey and Potvin, 2000). As stated by Wittebolle (2009) unevenness could block the rapid response of a community to a particular stress if the dominant species are not resistant to this stress. It is reported that even communities can recover their function more easily, provided with sufficient time (Wittebolle, 2009). The higher similarity in soil properties of the organic soil and the organic growing medium may also explain that no major differences in bacterial community structure were found between SOILANIMAL and SOILPLANT and GBOF and GBFISH, respectively, indicating that soil or growing medium are major discriminants of the microbial community composition. Amplicon sequencing showed that differences in the rhizosphere bacterial community composition were mainly impacted by fertilizer within soilless systems, while the opposite occurred in soil. PLFA results showed GBOF showed a positive correlation with ammonium, the total biomass, chloride, pH, magnesium and phosphorous, while GBFISH was positively correlated with fungal FAME marker 18:2 and fungal FAME marker 18:3 and nitrate. SOILPLANT, SOILANIMAL were positively correlated with AMF (arbuscular mycorrhizal fungi), gram-negative bacteria, the bacteria to fungi ratio, calcium, sulfate, phosphorous, gram-positive bacteria, pH, sodium, and chloride. The soilless culture systems GBOF and GBFISH, however, showed a positive correlation with protozoa and the fungal fatty acid methyl esters (FAME) marker 18:2 and 18:3 and it was negatively correlated with the Gram-positive bacteria and the Actinomycetes. The use of blood meal and malt sprouts in the organic soil was positively correlated to AMF, Gram-negative and Gram-positive bacteria. Fungal FAME marker 18:1, 18:2, and 18:3 was negatively influenced by the application of blood meal and malt sprouts. These results indicated that fertilizer incorporation increased disturbance in fungal communities in both cultivation systems. Overall the soil culture system seems to be positively correlated with Gram negative, Gram positive bacteria and AMF. Arbuscular mycorrhizal fungi (AMF) are ubiquitous organism that influence soil fertility through the enhancement of chemical, biological and physical content. Actinomycetes are an important class of soil microorganisms that are known to decompose complex polymers and cycle more recalcitrant soil organic matter. These results indicate that soil is a highly dynamic environment, where bacterial communities are rapidly adapting, while community characteristics of bacteria inhabiting growing medium stay uniform over time despite fertilizer application.

The four tomato cultivation systems were managed independently from each other and we demonstrated that it is possible to grow tomatoes in soilless culture and in soil based systems. Similar plant performance was observed in soilless culture systems and soil based system and yield was the highest with the aquaponics-derived fertilizer, but showed on the contrary the lowest average fresh and dry plant weight. This is in agreement with the study of Heeb (2005); Heeb et al. (2005a), and Heuvelink (2018). The yield per surface unit for GBOF and SOILANIMAL and SOILPLANT was quite similar for the three treatments. However, when yield is calculated per unit of available root volume, we found final cumulative yield of 3.1 kg tomatoes L^–1^ of growing medium and 2.8 kg tomatoes L^–1^ of growing medium for GBFISH and GBOF, respectively, while the soil based system produced approximately 0.5 kg tomatoes L^–1^ soil. Soilless culture systems possess a finite root volume, but they give complete control over water and the fertigation solution with a more precise composition and ratio of nutrients resulting in higher yields (Gruda, 2008). However, unbalanced water supply and high organic nitrogen supply rates will easily result in nutrient imbalance and further induce blossom end-rot (BER) of glasshouse tomatoes, which was reflected in the highest percentage of BER, i.e., 2.8% in the soilless culture system (Britto and Kronzucker, 2002; Heeb, 2005). Evolution of plant length (Figure 2) was quite similar between the four contrasting tomato cultivation systems, except for GBOF where plants were longer. From the experimental setup it is clear that we have different forms (organic nitrogen, ammonium and nitrate – nitrite is not considered) and concentrations of nitrogen in the four tomato cultivating systems. Plants can assimilate these different kind of nitrogen forms (Näsholm et al., 2009; Marschner, 2011). Mineralization rates are not equal for the different organic fertilizers, such as the blood meal, malt sprouts and the organic fertilizers used in combination with GBOF (Stadler et al., 2006; Dion et al., 2020). In addition, mineralization first releases ammonium, that is then converted in nitrate during nitrification, so the concentration of nitrate depends on both the concentration of ammonium and the ammonia and nitrite oxidation rate (Boudsocq et al., 2012). Our results show a higher ammonium concentration in combination with GBOF, indicating a higher ammonification rate or a lower nitrification rate. Ammonium uptake and assimilation are less energy demanding than nitrate uptake and assimilation, indicating a competitive advantage for plants with a high ammonium absorption capacity. High ammonium concentrations, however, can also cause severe toxicity symptoms (Britto and Kronzucker, 2002). This ammonium toxicity may jeopardize the energetic advantage of taking up ammonium rather than nitrate. Furthermore, ammonium is known for its abiotic immobilization, while nitrate is highly mobile and can lead to leaching losses. These physical limitations, energetic costs and competition with the soil microorganisms make these systems highly dynamic and almost unpredictable.

Our study highlights some limitations of previous studies and enlarges our awareness about the impact of soilless culture and soil based culture systems and different fertilizers on the below ground microbiology in tomato cultivation systems, because (1) soil and soilless culture systems were assessed at the same time, (2) amplicon sequencing and PLFA were similarly used as supplementary techniques to allow quantification of microbial biomass and (3) nutrient and N dynamics and (4) plant performance was followed and assessed over time. Community composition in soil plus either fertilizer remained unaltered over time, excepting on the first time point of soil supplemented with plant-derived fertilizer, when the relative abundance of *Mycoplasma* was significantly increased. Unclassified bacteria and C*lostridium* were taxa exclusively present when soil was amended with animal manure, while the relative abundance of *Bacillus* increased when plant-derived fertilizer was added. On the contrary, community composition of soilless systems was impacted by fertilizer and *Mycoplasma*, *Rhizomicrobium*, *Nocardioides* and *Devosia* were only detected in the aquaponics, while *Pseudomonas*, *Dyella* and *Flavobacterium* increased relative abundance in soilless systems with organic fertilizer. Thus, differences in the rhizosphere bacterial community composition were mainly impacted by fertilizer within soilless systems, while the opposite occurred in soil.

To the best of our knowledge, the presented study is the first study that carried out an in-depth observation of four contrasting tomato cultivation systems during one growing season on the composition of the bacterial and the fungal microbiome by using amplicon sequencing and PLFA. At the start of a soilless culture, which is considered as a microbial vacuum (Postma et al., 2000) and in contrast to an organic soil based systems, a microbial community promptly occupies the growing medium (Grunert et al., 2016a), the fertigation solutions and the rhizosphere of the cultivated plants (Grunert et al., 2016b, 2019). The microbial community composition is affected by the type of growing medium (Grunert et al., 2016a), the fertilizer used (Grunert et al., 2019) and plant species (Vallance et al., 2010). For the soil and growing media sampling soil was taken close to the rootstock. As these zones were fully colonized it might be the case that the “bulk” soil samples for the microbial community analysis can be considered as rhizosphere soil samples. Due to this experimental restriction and differences in root density between the four contrasting tomato cultivation systems, this might impact the microbiome present. Cultural methods have been used to characterize this microbial community, but molecular based techniques such as amplicon sequencing is known to give solid data on microbial taxonomy, species richness, evenness and diversity, while PLFA analysis add completing information on total biomass, and biomass per specific group.

## Conclusion

In the current study, we demonstrated and confirmed that it is possible to grow tomatoes in soilless culture systems in combination with organic fertilizers or in an aquaponics systems and in an organic soil fertilized with plant, i.e., malt sprouts or animal derived, i.e., blood meal during a whole season (321 DAS). We compared the bacterial and fungal community structure of soil and soilless culture systems for the cultivation of tomatoes with two complementary molecular techniques. We showed that the culture system impacted bacterial community characteristics and showed opposite trends. The individual observations are not new and confirm the results of earlier studies, however, this is the first study to show that bacterial and fungal diversity under long term fertilization with malt sprouts and blood meal show a similar behavior in soil, while soilless culture systems show higher responsiveness to fertigation management. This work contributes to a better understanding of the general principles governing fungal and bacterial community structure and adaptation in soil and soilless culture systems, and is widely applicable to sustainable agriculture and horticulture. This work also addresses a knowledge gap between soil based and soilless culture systems.

## Data Availability Statement

The sequencing data generated for this study can be found in NCBI using accession number PRJNA574435 (https://www.ncbi.nlm.nih.gov/bioproject/PRJNA574435).

## Author Contributions

OG, EH-S, SB, and NB conceived and designed the experiments. OG performed the experiments. EH-S processed the Illumina libraries, performed the data mining, statistical analysis, interpretation, and figure and table preparation of the 16S rRNA amplicon sequencing results. OG completed the statistical data processing of the physicochemical variables measured. NB and SD contributed with the reagents, materials, analysis tools, and revisions of the manuscript. Contributed equally co-authors contributed equally to revisions of the manuscript. All the authors contributed to the article and approved the submitted version.

## Conflict of Interest

OG was employed by the company Greenyard Horticulture Belgium at the time this research was carried out, and is now employed by Aphea.bio. The remaining authors declare that the research was conducted in the absence of any commercial or financial relationships that could be construed as a potential conflict of interest.

## Supplementary Material

Supplementary Figure 1 **(A)** Sample collection and analysis procedure. Slabs for the soilless culture system were placed in gutters, each gutter contained 6 slabs and each slab 5 plants. The two outer rows and outer slabs of each block were not selected, because of possible interactions with the adjacent rows and to avoid side effects. One slab with 5 consecutive plants in the soilless culture system and 5 consecutive plants in the soil based system were considered as an experimental unit. Among all treatments the tomato plants were placed consecutively with an interspacing of 0.47 m and an in row interspacing of 0.8 m. For the soilless culture system and soil-based system five experimental units were randomly selected from the different compartments. Samples of the different experimental units were collected at different time points during the growing season and at the start of the experiment. Ten subsamples from each experimental unit were collected, pooled, homogenized and treated as a single sample. **(B)** At each time point, samples were taken from 5 fixed experimental units of each GBOF, GBFISH, SOILANIMAL and SOILPLANT, including root material. Samples from the soil were taken with an auger in the 0–10 soil profile and from each experimental subunit 10 subsamples were taken. Each sample contained 200 *g* soil or growing medium and was divided into homogenous subsamples: one subsample was used for chemical analyses (100 g) and water content (50 g), one subsample was immediately after sampling stored on dry ice, preserved at −80°C and used for molecular microbial community analysis (50 g). The ammonium and the nitrogen content, the pH and the electrical conductivity (EC) in the 0–10 cm organic soil layer and in the growing medium were taken at the start 55 DAS(T0), 68 DAS (T1), 83 DAS (T2), 113 DAS (T3), 146 DAS (T4), 172 DAS (T5), 221 DAS (T6), and 321 DAS (T7). Samples for microbial community analysis were taken at 8 different timepoints, i.e., 55 DAS, 68 DAS, 83 DAS, 113 DAS, 146 DAS, 172 DAS, 221 DAS, and 321 DAS. Samples for PLFA analyses were collected at 4 different time points, i.e., 55 DAS, 83 DAS, 221 DAS and 321 DAS. Briefly, for the PLFA analyses the soil and growing media were freeze-dried using a modified technique (Bligh and Dyer, 1959). Whole plants were harvested, chopped and samples from stem and leaves without tomatoes were collected for analysis at 221 DAS and 321 DAS.

Supplementary Figure 2 Overview of the cumulative water dosage (L per m^2^) for the different tomato cultivating systems.

Supplementary Table 1 Overview of the chemical composition of SOILPLANT and SOILANIMAL before the start of the experiment (8/1/2015) and throughout the whole experimental period. *n* = 1. As bulk density 1.25 t ha^–1^ (Vlaamse zandstreek; Arthur et al., 2011) was chosen for the 0.3 m top soil layer.

Supplementary Table 2 Overview of the chemical composition of the different fertilizers used. “−” means that the elements was not analyzed or specified. “^∗^” means according to the specifications of the supplier. “^∗∗^” means that the chemical composition was actually analyzed.

Supplementary Table 3 Overview of the fertilizers used and the total amount used for the different treatments. GBOF, soilless culture system with organic growing medium and organic fertilizer. GBFISH, soilless culture system with organic growing medium and fish. SOILANIMAL, organic soil with animal-derived material as fertilizer; and SOILPLANT, organic soil with plant-derived material as fertilizer.

Supplementary Table 4 Overview of the chemical composition of the four different organic fertilizers (Nutrikali, ANTYS MgS, Biosyr and SP). “–“: means that this element was not determined in the fertilizer.

Supplementary Table 5 Correlations between microbial community composition and chemical characteristics in four different tomato cultivating systems across time points. indicated by the Multiple Factor Analysis. Dimensions of the MFA can be described by the categorical variables included in the analysis. For each categorical variable (growing medium and time point). *A* one-way analysis of variance was performed with the coordinates of the samples on the axis. explained by the time point or growing medium type. Then, for each level of the category (i.e., time point 1. time point 2 or time point 3 or growing medium GB). *A* Hotelling T^2^-test was used to compare the average of the category with the general average (using the constraint P i αi = 0. αi = 0). For instance. the coordinates of the relative abundance of family “x” at GB at time point 1 were compared with the average coordinates of the relative abundance of family “x” in GB. The P*-*value associated to this test is transformed to a normal quantile to assess whether the mean of the category is significantly less or greater than 0. Negative values indicate negative correlations.
