# Supplementary material for: In-Depth Observation on the Microbial and Fungal Community Structure of Four Contrasting Tomato Cultivation Systems in Soil Based and Soilless Culture Systems
Source: Front Plant Sci. 2020 Nov 5;11:520834. doi: 10.3389/fpls.2020.520834 (PMC7674179; doi:10.3389/fpls.2020.520834)
Supplement: Supplementary Figure 1 — (A) Sample collection and analysis procedure. Slabs for the soilless culture system were placed in gutters, each gutter contained 6 slabs and each slab 5 plants. The two outer rows and outer slabs of each block were not selected, because of possible interactions with the adjacent rows and to avoid side effects. One slab with 5 consecutive plants in the soilless culture system and 5 consecutive plants in the soil based system were considered as an experimental unit. Among all treatments the tomato plants were placed consecutively with an interspacing of 0.47 m and an in row interspacing of 0.8 m. For the soilless culture system and soil-based system five experimental units were randomly selected from the different compartments. Samples of the different experimental units were collected at different time points during the growing season and at the start of the experiment. Ten subsamples from each experimental unit were collected, pooled, homogenized and treated as a single sample. (B) At each time point, samples were taken from 5 fixed experimental units of each GBOF, GBFISH, SOILANIMAL and SOILPLANT, including root material. Samples from the soil were taken with an auger in the 0–10 soil profile and from each experimental subunit 10 subsamples were taken. Each sample contained 200 g soil or growing medium and was divided into homogenous subsamples: one subsample was used for chemical analyses (100 g) and water content (50 g), one subsample was immediately after sampling stored on dry ice, preserved at −80°C and used for molecular microbial community analysis (50 g). The ammonium and the nitrogen content, the pH and the electrical conductivity (EC) in the 0–10 cm organic soil layer and in the growing medium were taken at the start 55 DAS(T0), 68 DAS (T1), 83 DAS (T2), 113 DAS (T3), 146 DAS (T4), 172 DAS (T5), 221 DAS (T6), and 321 DAS (T7). Samples for microbial community analysis were taken at 8 different timepoints, i.e., 55 DAS, 68 DAS, 83 DAS, 113 DAS, 146 DAS, 17 [file Data_Sheet_1.PDF]

Effect of long-term fertilization in soil and soilless culture of tomatoes on the microbial and fungal community structure

Oliver Grunert[1, 2], Emma Hernandez-Sanabria[1], Saskia Buysens[3], Stefaan De Neve[4], Marie-Christine Van Labeke[5], Dirk Reheul[5], and Nico Boon[1*].

[1]Center for Microbial Ecology and Technology (CMET), Ghent University, Coupure Links 653, 9000 Gent, Belgium.

[2]Aphea.Bio, Technologie Park 21, Zwijnaarde, 9052 Belgium.

[3] PCG - Vegetable Research Centre, Karreweg 6, 9770 Kruishoutem, Belgium

[4]Faculty of Bioscience Engineering, Department of Environment, Ghent University, Coupure Links 653, 9000 Gent, Belgium.

[5] Faculty of Bioscience Engineering, Department of Plants and Crops, Ghent University, Coupure Links 653, 9000 Gent, Belgium.

*** Correspondence:**

Nico Boon
Nico.Boon@UGent.be

Keywords: tomato, soilless culture systems, fertilizer, growing medium, soil

Supplementary table 1: Overview of the chemical composition of soilplant and soilanimal before the start of the experiment (8/1/2015) and throughout the whole experimental period. n=1. As bulk density 1.25 t ha^{-1} (Vlaamse zandstreek; [Arthur et al. [58]]) was chosen for the 0.3 m top soil layer.

**Treatment**	**Date analysis**	**NO_3-N kg ha^{-1}**	**NH_4-N kg ha^{-1}**	**(N-mineral)**
SOILANIMAL	8/01/2015	243	< 4	247
SOILPLANT	8/01/2015	196	< 4	200
SOILANIMAL	3/2015	68	<4	72
SOILPLANT	3/2015	97	9	106
SOILANIMAL	5/2015	25	<4	29
SOILPLANT	5/2015	6	7	13
SOILANIMAL	6/2015	18	<4	22
SOILPLANT	6/2015	42	8	50
SOILANIMAL	27/7/2015	16	7	23
SOILPLANT	27/7/2015	17	18	35
SOILANIMAL	4/11/2015	10	<4	14
SOILPLANT	4/11/2015	9	<4	13

Supplementary table 2: Overview of the chemical composition of the different fertilizers used. “-“ means that the elements was not analyzed or specified. “” means according to the specifications of the supplier. “**” means that the chemical composition was actually analyzed.*

**Fertilizer**	**Composition (%)**								
	**N_{total}**	**NO_3^--N**	**NH_4^+-N**	**P_2O_5**	**K_2O**	**MgO**	**CaO**	**Cl**	**SO_3**
Blood meal*	14	-	-	0	0	-	-	-	-
Patentkali*	-	-	-	-	30	10	-	-	42
Magnesium sulphate*	-	-	-	-	-	16	-	-	32
Malt sprouts*	3	-	-	0	0	-	-	-	-
Antys MgS**	0	-	0	0	0	9	0	0	18
Biosyr**	9.35	-	0.53	3.96	9.08	0.11	3.98	0.68	10.9
Nutrikali**	2.4	-	0.1	0.3	4.9	0.1	0.1	1.1	1.1
SP**	0	-	0	0.001	58.6	0	0	0	0
Calcium chloride 33 %*	0	-	0	0	0	0	16.5	21	0
Calsal vlb 51%*	8.7		-	-	-	-	8.7	-	-
Amnitra vlb 51%*	18	9	9	-	-	-	-	-	-
Potassium nitrate*	13	13	-	-	45	-	-	-	-
Magnesium sulphate*	-	-	-	-	-	16	-	-	32
Monopotassium phosphate*	-	-	-	52	34	-	-	-	-
Potassium sulphate *	-	-	-	-	50	-	-	-	45

Supplementary table 3: Overview of the fertilizers used and the total amount used for the different treatments. GBOF = soilless culture system with organic growing medium and organic fertilizer. GBFISH = soilless culture system with organic growing medium and fish. SOILANIMAL= organic soil with animal-derived material as fertilizer and SOILPLANT= organic soil with plant-derived material as fertilizer

**Treatment**	**Type of fertilizer used**	**Total amount of fertilizer used (g m^{-2})**
GBFISH	Calsal vlb 51% ($Ca(NO_3)_2$)	1855
	Amnitra vlb 51% (NH_4NO_3)	557
	Potassium nitrate (KNO_3)	959
	Magnesium sulphate ($MgSO_4$)	279
	Monopotassium phosphate (KH_2PO_4)	368
	Potassium sulphate (K_2SO_4)	288
GBOF	Antys MgS	827
	Biosyr	1292
	Nutrikali	1870
	SP	404
	$CaCl_2$	921
	Libremix	49
SOILANIMAL	Blood meal	180
	Patentkali	163
SOILPLANT	Malt sprouts	1001
	Patentkali	163

37 *Supplementary table 4: Overview of the chemical composition of the four different organic fertilizers*
38 *(Nutrikali. ANTYS MgS. Biosyr and SP). "-": means that this element was not determined in the*
39 *fertilizer*

	**Nutrikali**	**ANTYS MgS**	**Biosyr**	**SP**
Total Nitrogen (%)	2.35 ± 0.1 2	-	9.35 ± 0.23	-
Organic nitrogen (%)	2.25 ± 0.11	-	8.81 ± 0.22	-
NH_4^+-N (%)	0.056 ± 0.003	-	0.532 ± 0.027	-
NO_3^--N (%)	0.042 ± 0.002	-	0.012 ± 0.001	-
P_2O_5 (%)	0.257 ± 0.013	-	3.96 ± 0.20	0.0007 ± 0.0001
K_2O (%)	4.93 ± 0.25	-	9.08 ± 0.23	58.6 ± 1.5
CaO (%)	60.113 ± 0.006	-	3.98 ± 0.20	-
MgO (%)	0.121 ± 0.006	0.725 ± 0.036	0.110 ± 0.005	-
SO_3^{2-} (%)	1.11 ± 0.06	1.28 ± 0.06	10.9 ± 0.3	-
Na_2O (%)	1.97 ± 0.10	-	0.836 ± 0.042	-
Cl (%)	0.839 ± 0.042	-	0.676 ± 0.034	-
Organic matter (%)	0.839 ± 0.042	-	57.4 ± 1.4	-

Supplementary table 5: Correlations between microbial community composition and chemical characteristics in four different tomato cultivating systems across time points. indicated by the Multiple Factor Analysis. Dimensions of the MFA can be described by the categorical variables included in the analysis. For each categorical variable (growing medium and time point). a one-way analysis of variance was performed with the coordinates of the samples on the axis. explained by the time point or growing medium type. Then. for each level of the category (i.e. time point 1. time point 2 or time point 3 or growing medium GB). a Hotelling T^2-test was used to compare the average of the category with the general average (using the constraint P i αi = 0. αi = 0). For instance. the coordinates of the relative abundance of family "x" at GB at time point 1 were compared with the average coordinates of the relative abundance of family "x" in GB. The P value associated to this test is transformed to a normal quantile to assess whether the mean of the category is significantly less or greater than 0. Negative values indicate negative correlations.

Dimension	Variance	Descriptor	Estimate (R^2)	P value	Taxon and or chemical characteristics	Correlation	*P* value
					K	0.9505387	<0.0001
					Protozoa	0.882805	<0.0001
					EC	0.8713766	<0.0001
		GBOF	1.5492	0.00261	Fungi.18.3	0.7156968	1E-07
		GBFISH	1.451668	0.015256	Na	0.7015117	3E-07
					Total	0.6796157	0.000001
					Ca	0.6779943	1.1E-06
					P	0.6499159	4.3E-06
DIM 1					NO_3^-–N	0.6348602	8.3E-06
					Fungi.18.2	0.5960548	3.92E-05
					Cl	0.548466	0.000205
					Mg	0.4452108	0.003536
					SO_4^{2-}	0.4374311	0.004237
					Actinomycetes.	-0.3618954	0.020071
					pH(H2O)	-0.6273887	1.13E-05
					Bacteria Fungi18.2 ratio.	-0.6355404	8.00E-06
					Gram-positive bacteria	-0.7987867	<0.0001
DIM 2					AMF	0.729531	1E-07
					Gram-negative bacteria	0.7288208	1E-07

				Bacteria Fungi ratio.18.2.	0.670518	1.6E-06
	tpt4	1.178248	0.014011	Ca	0.5255753	0.000418
	SOILPLANT	0.8708145	0.016697	SO_4^{2-}	0.4707461	0.001895
	SOILANIMAL	0.7376762	0.103984	P	0.4189329	0.006407
				Gram-positive bacteria	0.4164783	0.006757
				pH(H2O)	0.4021037	0.009157
				Na	0.3751969	0.015642
				Cl	0.3520146	0.024004
				Fungi.18.3	-0.3528578	0.023645
				NH_4^+-N	-0.517281	5.34E-04
				Actinomycetes	-0.5900243	4.91E-05
				Fungi.18.2	-0.6236232	1.33E-05
				Fungi.18.1	-0.8010289	<0.0001
DIM 3				NH_4^+-N	0.675832	1.2E-06
				Total	0.6403078	6.5E-06
	GBOF	1.27019	5.96E-05	Cl	0.5425307	0.000248
				pH(H2O)	0.4863632	0.001263
				Mg	0.3706436	0.017054
				P	0.3569491	0.021966
				Fungi.18.2	-0.446345	0.003443
				Fungi.18.3	-0.49035	1.14E-03
				NO_3^-—N	-0.722229	1.00E-07
				Actinomycetes	0.5783302	7.49E-05
DIM 4	SOILANIMAL	-0.6524775	0.005355	Gram-negative bacteria	0.5103223	0.000653
				SO_4^{2-}	0.4863936	0.001262
				NH_4^+-N	0.4423815	0.003778
	tpt3	0.4160831	0.051796	Mg	0.3565815	0.022113
DIM 5				Gram-negative bacteria	0.3443766	0.027467
				Cl	-0.313677	0.045812

	Na	-0.320351	0.041154
	Fungi.18.1	-0.347512	0.025998
	P	-0.39069	0.011552

9a SOILLESS CULTURE

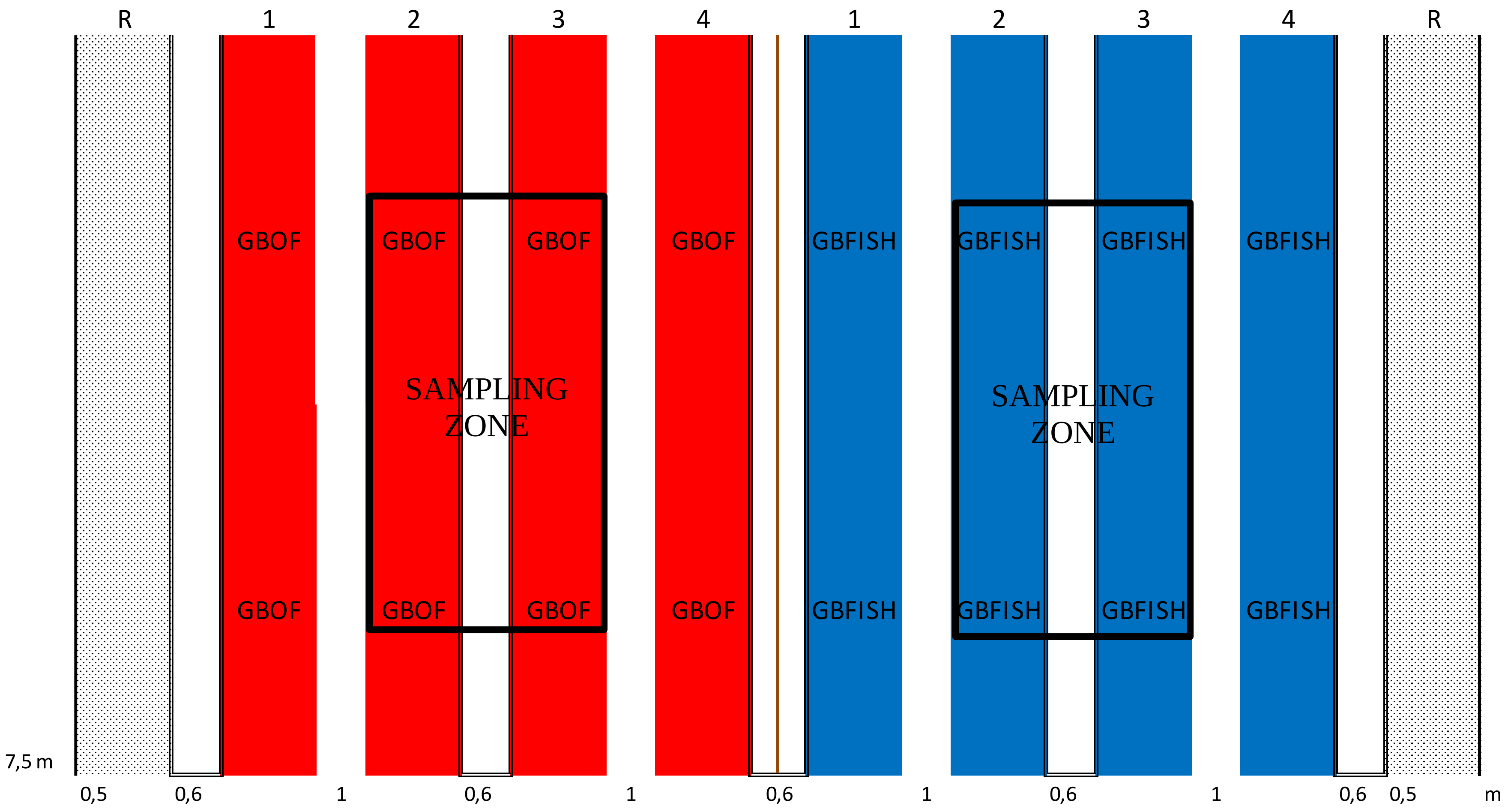

9b SOILANIMAL

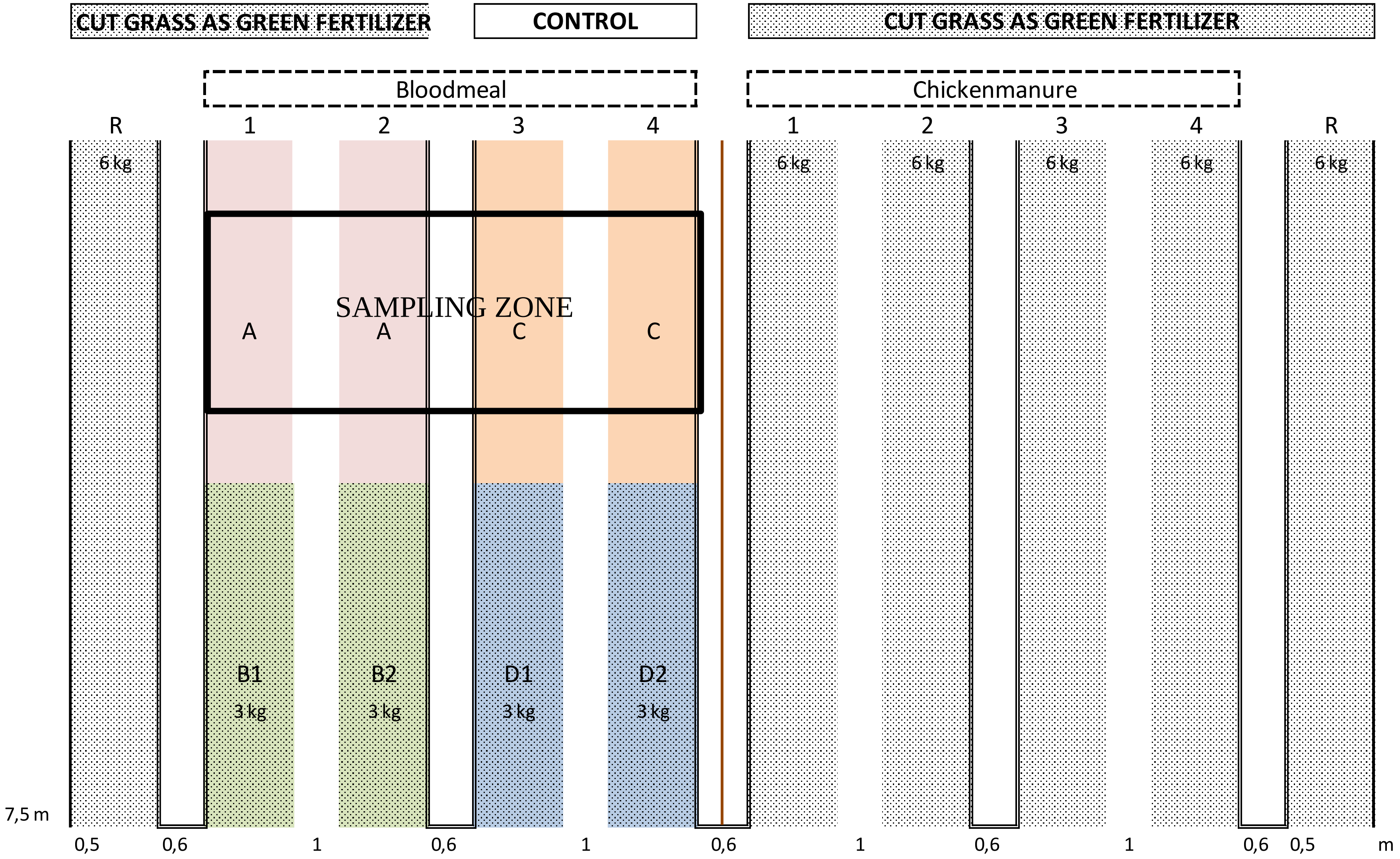

58

9c SOILPLANT

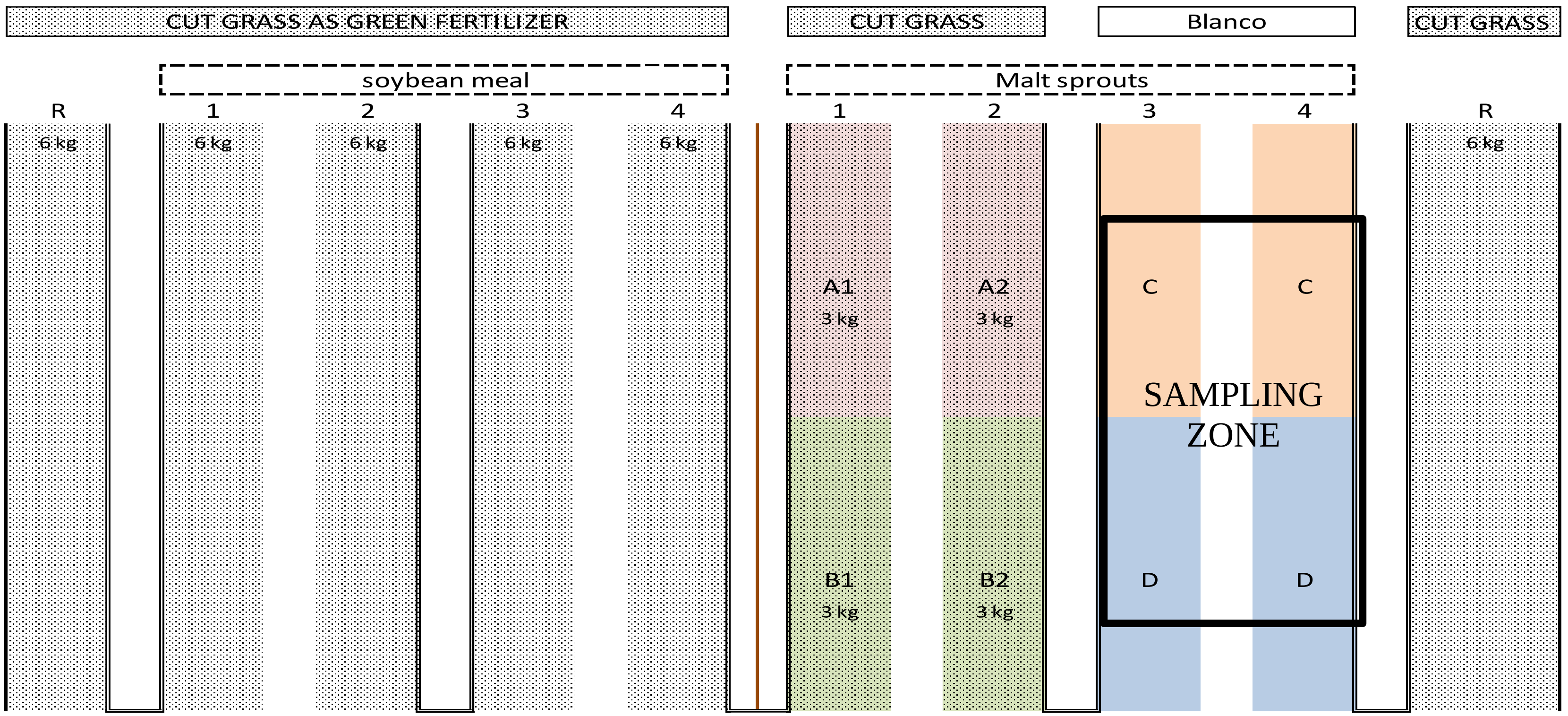

Supplementary figure 1: Overview of the experimental set-up. The glass house was divided into three compartments (S91 = 9a GBOF and GBFISH. S92=9b soilanimal and S93 = 9c soilplant) with a surface of 80 m². S91 was subdivided into two part. i.e. red color = GBOF and blue color = GBFISH; S92 was organic soil with animal (blood meal) derived material and S92 was the organic soil with plant-derived material (malt sprouts). In S92 and S93 the previous cultures were tomato in 2014. pepper in 2013 and cucumber in 2012. R= outer rows. Plant density was the same for all the treatment 2.65 plants m^{-2}. Samples were taken in the sampling zone with 40 plants per sampling zone.

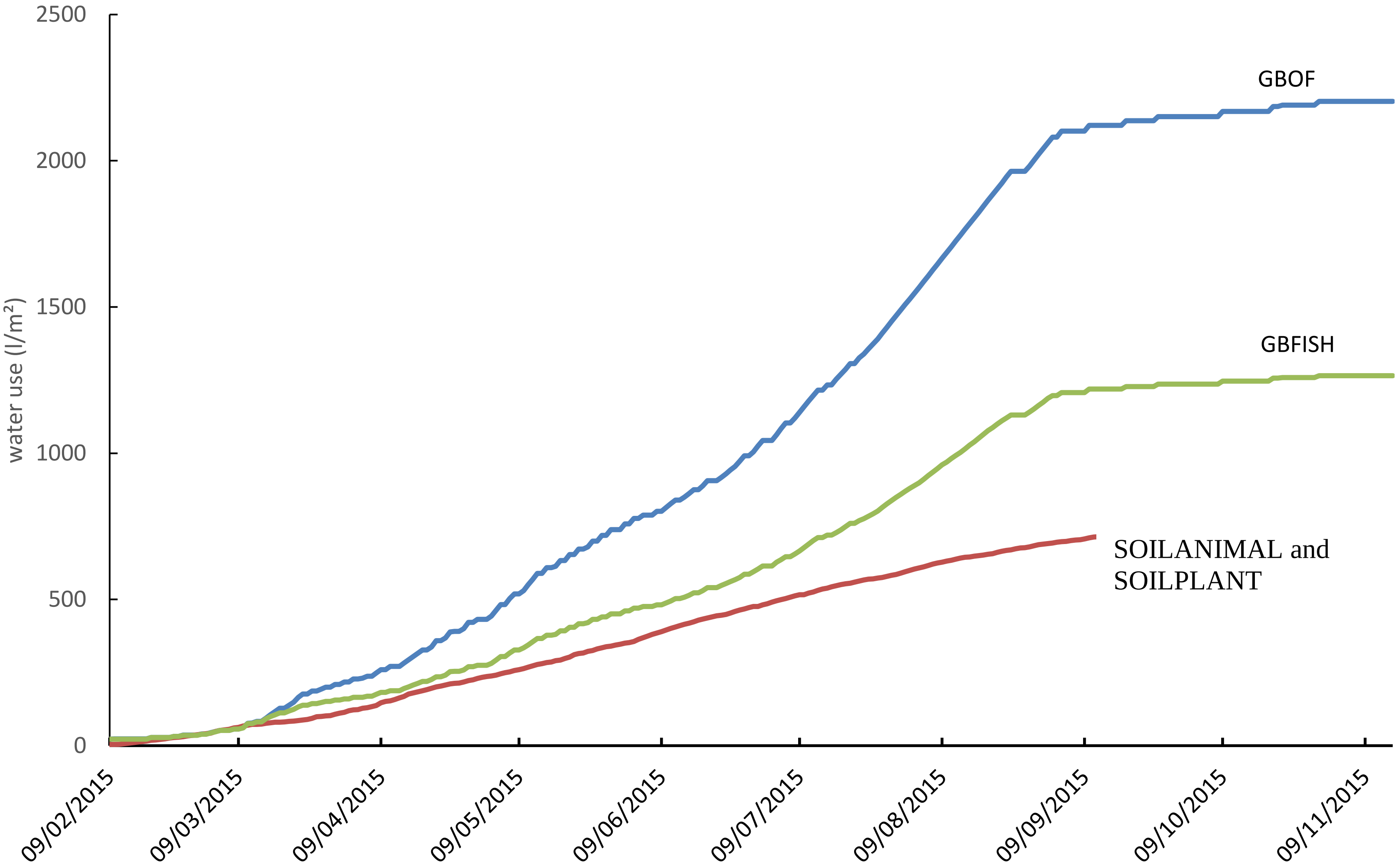

Supplementary figure 2: Overview of the cumulative water dosage (L per m²) for the different tomato cultivating systems.
